# Morphology-agnostic humanoid retargeting via perception-motivated graph similarity

**DOI:** 10.3389/fnbot.2026.1814977

**Published:** 2026-07-23

**Authors:** Chaojie Fu, Chengkai Su, Lei Jiang, Kaixin Lan, Yongbin Jin, Hongtao Wang

**Affiliations:** 1 The Center for X-Mechanics, Zhejiang University, Hangzhou, Zhejiang, China; 2 The State Key Laboratory of Fluid Power and Mechatronic System, Zhejiang University, Hangzhou, Zhejiang, China; 3 The Institute of Applied Mechanics, Zhejiang University, Hangzhou, Zhejiang, China; 4 ZJU-Hangzhou Global Scientific and Technological Innovation Center, Zhejiang University, Hangzhou, Zhejiang, China

**Keywords:** character animation, humanoid robot, motion capture data, motion retargeting, similarity metric

## Abstract

Quantifying motion similarity, despite its inherently subjective nature, is a foundational problem for humanoid motion transfer and control across different embodiments. Drawing from cognitive studies revealing that human motion similarity judgments are strongly influenced by spatial relationships among body parts and proximal contacts, we develop a graph-based representation that effectively encapsulates both features. This representation enables the definition of a robust similarity metric through graph distance computations. The proposed metric emphasizes spatial, especially proximal, relationships between body parts, facilitating motion retargeting that preserves these perceptually motivated relational cues across humanoid embodiments with shared semantic body parts and varying Degree of Freedom (DoF) configurations and body proportions. For quantitative retargeting evaluation, we introduce an order-preserving spatial similarity metric that measures how consistently inter-joint distance rankings are preserved between source and target motions.

## 1 Introduction

Accurately transferring and evaluating human motion is a fundamental problem in humanoid robotics, where whole-body behavior must be represented and compared across systems with different embodiments. Motion similarity refers to the extent to which two motions resemble each other in terms of kinematics, dynamics, and expressive qualities. However, quantifying motion similarity remains a significant challenge due to its inherently subjective nature and the complexity of aligning diverse motion features. This challenge is also relevant to computer graphics and animation, but is particularly critical in humanoid motion retargeting, where motion captured from one performer must be adapted to robots with different skeletal structures and proportions while preserving perceptually important similarities.

Historically, animators relied on laborious keyframe techniques to create human-like motion. The introduction of motion capture (mocap) technology greatly streamlined this process by recording realistic movements directly from performers. However, when such motion data must be applied to targets with different morphologies, a process known as motion retargeting, the difficulty of accurately preserving motion similarity re-emerges. Automating efficient and scalable motion retargeting benefits from a similarity metric motivated by perceptual findings on motion similarity.

Although human perception of motion similarity remains highly subjective and intuition-driven, some cognitive studies suggest that pairwise spatial relationships Tang et al., (2008) and proximity, especially self-contacts, Basset et al. (2022) critically influence perceptual similarity judgments. Johansson (1973) and Blake and Shiffrar (2007)'s pioneering point-light motion experiments revealed that it is not simply the motion of individual points but the structured spatial organization of joints that enables the visual system to identify biological movement. Recognizing that these observations highlight pairwise relationships, we adopt an inter-joint graph representation related to prior pairwise-relational and interaction-graph formulations to capture spatial and proximal relationships between body parts and the environment. In this work, we extend this relational representation for humanoid retargeting by introducing an explicit ground vertex and a proximity-weighted graph distance, which together define the similarity objective used for retargeting and the metric used for spatial-relationship-preservation analysis. By preserving relational features on semantically aligned humanoid landmarks, this similarity metric supports motion transfer across humanoid-like skeletons with varying Degree of Freedom (DoF) configurations and body proportions.

We formulate motion retargeting as a constrained optimization problem for graph distance and implement the proposed framework for retargeting diverse human motions from standard mocap databases, including CMU (Carnegie Mellon University, 2012) and AMASS (Mahmood et al., 2019), to humanoid robots. Our approach focuses on humanoid-like skeletons with shared semantic body parts, while allowing differences in DoF configurations and body proportions. To evaluate how well this approach preserves spatial relationships across body proportions, we manually construct hundreds of robots with different body proportions and execute retargeting with our method. We further employ sensitivity analysis to examine the role of proximal weighting in the similarity metric.

Specifically, this work makes three contributions. First, we formulate a proximity-weighted graph similarity metric for humanoid retargeting by augmenting an inter-joint vector graph with a dedicated ground vertex and edge weights based on the closeness of body parts. Second, we formulate motion retargeting as a constrained optimization over joint angles using this similarity as the objective, enabling transfer across humanoid-like skeletons with shared semantic body parts and different DoF configurations and body proportions. Third, through large-scale retargeting experiments, spatial-relationship-preservation analysis, and sensitivity analysis, we evaluate the proposed framework on diverse humanoid motions and target bodies.

The structure of this paper is as follows. Section 2 reviews related work. Section 3 presents the graph representation and retargeting formulation. Section 4 reports the experimental evaluation and analysis. Section 5 discusses the results and limitations. Section 6 summarizes the contributions and future directions.

## 2 Related work

This section reviews related studies in two areas. The first concerns motion representation and similarity metrics, from joint-based encodings to perceptually guided descriptors that emphasize spatial and proximal relationships. The second covers motion retargeting methods for adapting movements to characters or robots with varying structures, focusing on how different approaches preserve similarity.

### 2.1 Human motion representation and similarity

Human motion representation methods have evolved from basic joint-orientation representations to perceptually guided descriptors capturing spatial relationships, and finally to structure-preserving formulations that maintain local geometric consistency.

Early works (Lee et al., 2002; So and Baciu, 2005; Sempena et al., 2011) directly employed raw joint orientations as motion representations for similarity measurement and recognition. Although computationally simple, such representations often misalign with human perceptual judgments, remain sensitive to anthropometric differences and typically ignore contact semantics, limiting their robustness when source and target morphologies diverge. To bridge this gap, perceptually grounded methods incorporated statistically sampled human similarity ratings to identify components critical to human motion perception. Empirical studies (Tang et al., 2008; Basset et al., 2022; Harada et al., 2004; Cheng Chen et al., 2011) showed that humans tend to prioritize Cartesian joint coordinates over orientations.

Beyond isolated joint measures, Joint Relative Distances (JRD) were proposed to capture pairwise spatial relationships. Tang et al. (2008) identified limitations of simple Euclidean distances with respect to perceptual similarity, proposing a weighted JRD metric with confidence-based similarity evaluation. Basset et al. (2022) further demonstrated its ability to capture perceptually relevant conditions such as self-contact, which raw coordinates fail to reflect. Extending JRD from scalars to vectors, Chen et al. (2009) included directional inter-joint information, while Zhang Y. et al. (2023) constructed an Interaction Graph whose edges represent inter-joint vectors to enable multi-character motion retargeting.

Related work has also explored correlation-based similarity measures as evaluation tools rather than retargeting objectives. In particular, Randall et al. (2023) used Spearman-rank correlation to compare motion sequences while remaining robust to non-linear transformations and outliers. We build on this line of evaluation methodology in Section 4.2, where rank correlation is applied to spatial inter-joint-distance orderings.

Motivated by these findings, we adopt an inter-joint vector representation to capture the spatial and proximal cues reported as perceptually important, organized into a graph-based structure akin to the Interaction Graph (Zhang Y. et al., 2023). Building on this prior relational line, our method introduces an explicit ground vertex and a proximity-weighted graph distance for humanoid retargeting and spatial-relationship-preservation analysis.

### 2.2 Motion retargeting

Practical animation systems such as Autodesk MotionBuilder and Blender-based retargeting tools like Rokoko provide mature workflows for motion-capture editing and character-rig retargeting. We mention them as practical context, while focusing the review on algorithmic methods for morphology-varying humanoid retargeting.

We organize prior retargeting by (1) geometric/optimization, (2) learning-based, and (3) physics-aware methods.

#### 2.2.1 Geometric/optimization based retargeting

Classical inverse kinematics methods treat retargeting as solving intermediate joint poses for a kinematic chain given end-effector poses, optionally with task-priority schemes for redundancy resolution (Monzani et al., 2000; Kulpa et al., 2005). This frame-wise alignment remains attractive for its simplicity and real-time behavior but under-specifies contacts, often causing foot sliding, joint-limit violations, or self-penetration under large morphology changes (Gleicher, 1998; Kulpa et al., 2005).

Modern IK formulates retargeting as constrained optimization over weighted multi-task objectives (Araujo et al., 2025; Abdul-Massih et al., 2017; Bernardin et al., 2017; Kim et al., 2021; Cheynel et al., 2025), handling kinematic feasibility, joint limits, and collisions, and extending to multiple end-effectors (Abdul-Massih et al., 2017) and kinematic loops (Schumacher et al., 2021). Compared to classical IK, these formulations provide clearer constraint handling when transferring from mocap sources to robots; yet contacts remain implicit unless explicitly modeled in the objective or constraints (Gleicher, 1998; Kim et al., 2021).

Some optimization-based methods further target spatial-relationship preservation through Laplacian-deformation formulations. TMG (Li Y. et al., 2023) formulates motion generalization as a mesh-deformation problem on a mesh representation whose topology encodes spatial relationships among robot parts, and uses Laplacian coordinates to preserve these relationships within a single robot and across different robots.

Our approach adopts the geometric/optimization paradigm: we retarget motions by minimizing a proximity- and ground-aware similarity that is robust to differences in DoF configurations and body proportions within humanoid-like skeletons.

#### 2.2.2 Learning-based retargeting

Rather than relying solely on hand-crafted similarity metrics, learning-based methods learn task-aligned metrics from data, typically combining reconstruction or adversarial objectives with kinematic terms.

To generalize across heterogeneous skeletons, skeleton-agnostic embeddings separate morphology from motion semantics and decode to target structures (Zhang et al., 2022; Li T. et al., 2023; Raab et al., 2023), with some methods spanning human to non-human mappings (Li T. et al., 2023).

Because a skeleton's body parts and joints form a natural graph, graph neural networks (GNN) are widely used to enable skeleton-awareness (Aberman et al., 2020; Lee et al., 2023; Zhang et al., 2025). MeshRet (Ye et al., 2024) and R2ET (Zhang J. et al., 2023) further leverage skinned-body/mesh-aware representations and contacts/near-contact proximity to better preserve motion semantics during retargeting.

Concretely, methods either adopt a unified/canonical skeleton representation to embed motions into a shared latent space (Aberman et al., 2020; Lee et al., 2023), or learn dynamic graph transformations/structure-aware mappings between source and target skeletons (Zhang et al., 2025).

Unlike skeleton-aware learning methods, our approach does not learn cross-structure mappings; instead, it assumes semantically aligned humanoid landmarks and defines an analytical proximity- and ground-aware similarity metric for direct use in optimization.

#### 2.2.3 Physics-aware retargeting

Geometric retargeting often incurs foot sliding and base floating, hurting realism and control. Two remedies are common: contact-aware retargeting, which hard-codes foot/hand contacts to preserve support and avoid penetration (Al-Asqhar et al., 2013; Cheynel et al., 2025; Basset et al., 2020); and physics-based approaches, which simulate or control motions under dynamics (Zhang Y. et al., 2023; Reda et al., 2023; Al Borno et al., 2018; Rouxel et al., 2022; Grandia et al., 2023). Within this physics-aware setting, several closely related methods apply the interaction-mesh formulation directly to humanoid motion adaptation. The humanoid adaptation method of Nakaoka and Komura (2012) combines interaction-mesh-based spatial-relationship preservation with dynamic balancing, with a particular focus on close interactions between body parts. More recently, OmniRetarget (Yang et al., 2025) extends this interaction-preserving line to humanoid loco-manipulation and scene interaction, while generating retargeted references for downstream reinforcement learning. These methods, together with TMG, are closely related to our approach in their focus on preserving relational structure, although they use different representations.

## 3 Methods

This section explains how we represent motion, measure similarity, and use that similarity for motion retargeting. We start by abstracting each motion frame into semantically defined landmarks and their relationships, such as the relationship between the wrist and the knee. We also incorporate the ground so that important environmental interactions are represented in the same relational form. After expressing both the source and target motions in this shared relational form, we define a distance to quantify how these relationships differ across different embodiments. Guided by perceptual studies that highlight the importance of proximal relationships, we further refine this distance with proximity weighting. We then use the resulting weighted distance in the retargeting formulation.

### 3.1 Human motion graph

At a high level, each motion frame is represented as a graph whose vertices are semantically defined landmarks and whose edges encode relative positions between landmark pairs. We now formalize this representation.

**Figure 1:**
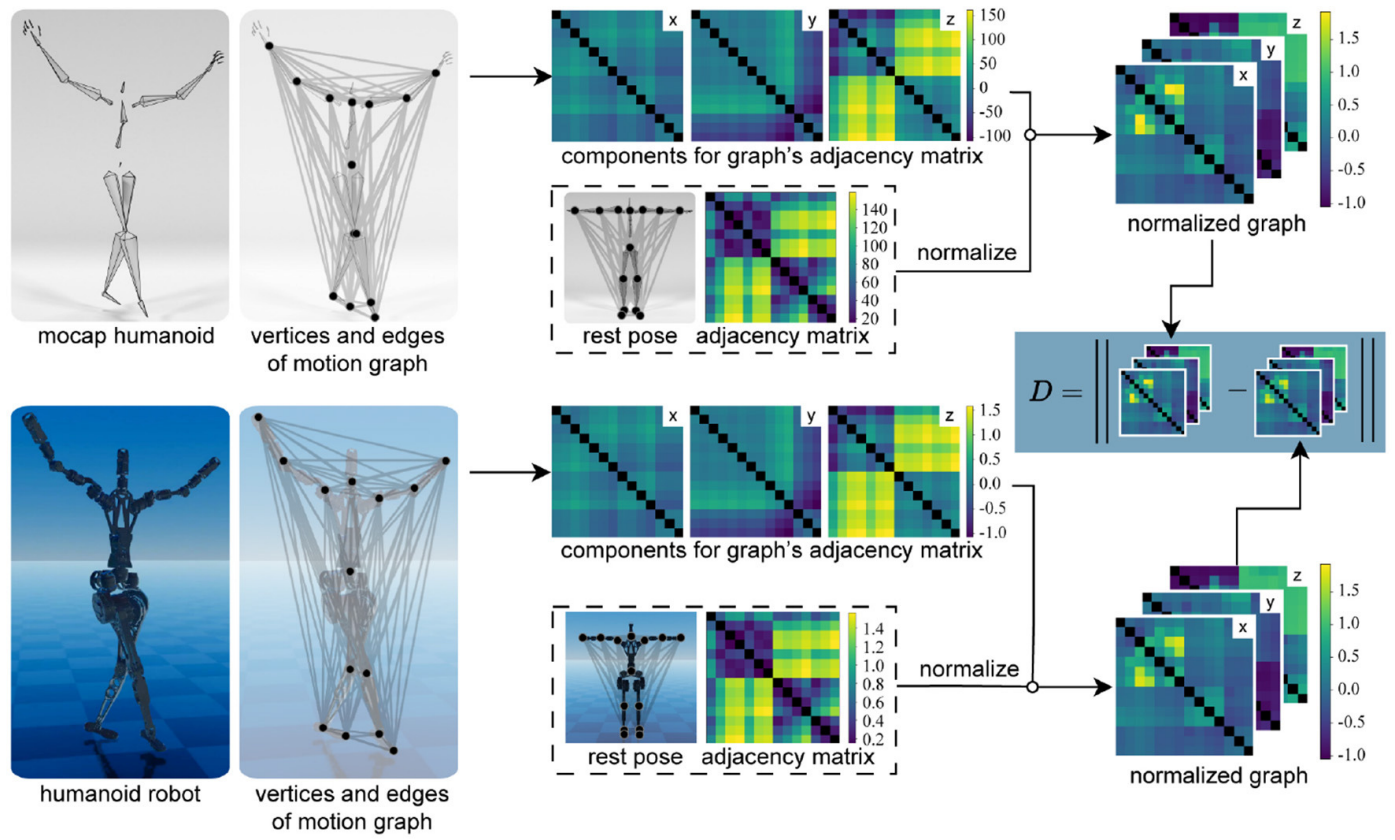
Schematic overview of the graph distance calculation pipeline for motion data from various sources. The upper panel shows mocap data in BVH format, and the lower panel depicts a kinematically similar motion executed by the GR-1 humanoid robot from Fourier Robotics. A unified motion graph representation is constructed through sequential steps: (1) defining anatomically critical landmarks, (2) computing inter-joint spatial vectors and (3) normalizing edges using the corresponding rest-pose lengths. The motion graphs are visualized as adjacency matrices, and the graph distance between source and target motions *D* is computed as the Frobenius norm of the edge differences, serving as a quantitative indicator of motion similarity.

We propose to represent human motion using a graph structure *G* = {*V, E*} as shown in Figure 1. The vertices $V=\left\{p_{i}\in {\text{IR}}^{3}|i=1,\ldots ,N\right\}$ are defined as the anatomically critical landmarks on the skeleton (solid dots in the left panel of Figure 1), whose positions are denoted by ***p*_*i*_**. For human-like characters, landmarks including the pelvis, chest, neck, head, shoulders, elbows, wrists, knees, ankles, and toes are commonly adopted as key points to comprehensively represent whole-body motion.

The graph edges (illustrated as gray lines in the left panel of Figure 1) are defined as three-dimensional spatial vectors pointing from one vertex to another. To address variations in skeleton sizes and proportions across motion data, we propose normalizing these vectors. More specifically, we normalize each spatial vector by the length of the corresponding vector in a canonical rest pose, defining the graph edges as normalized spatial vectors in Equation 1:


$$\begin{array}{l} E=\left\{E_{ij}=\left(p_{j}-p_{i}\right)/\|{\bar{p}}_{j}-{\bar{p}}_{i}\||\left(i,j\right)\in \Omega \right\} \end{array}$$ (1)

in which $\bar{p}$ is the correspondence of ***p*** in a rest-posed skeleton. The choice of rest pose is arbitrary, while common poses like T-pose and stand-pose are preferred. In this paper, we adopt the T-pose as the rest pose. Ω contains all feasible or curated pairs of vertex indices, here we define it as Ω = {(*i, j*)∣*i, j* = 1, 2, …, *N, i* ≠ *j*}.

Beyond skeletal vertices, we include objects from the external environment in the graph to further describe the relationship between the human and the environment. For human motion, the relationship between body parts and the ground is significant. Therefore, we introduce a dedicated ground vertex in the graph. Under the assumption of flat terrain, the vector between the ground vertex and a skeletal vertex can be defined as the normal vector between the ground plane and the key point. This definition of the human motion graph can be extended to non-flat terrains by incorporating a height map, thereby enabling a unified graph representation across uneven ground surfaces. In the non-flat-terrain demonstrations reported in Supplementary Video 2, this height-map form is used so that vertex-ground edges are computed with respect to the local terrain height rather than a single global ground plane.

During the construction of human motion graphs, both the source motion and the target body are reduced to the same set of semantically defined humanoid landmarks. In this representation, differences in raw DoFs are abstracted away because the graph is built from landmark positions and their pairwise relations rather than from a one-to-one comparison of every joint variable.

### 3.2 Graph distance and motion similarity

Once the source and target motions are expressed in the same landmark-based representation, we define their similarity through a distance between the corresponding motion graphs. Because the graph distance is computed from corresponding inter-landmark vectors, it compares both the relative distance magnitudes and directions between semantically corresponding landmarks. A smaller distance indicates better preservation of the source motion's spatial relationships, meaning that the retargeted pose more closely maintains the source pose's relative landmark distances and directions. We next formalize this distance.

To compute this distance, we represent each motion graph with a generalized 3D adjacency tensor of size [*N, N*, 3], where *N* is the number of vertices. Let *G*^*S*^ and *G*^*T*^ denote the source and target motion graphs, respectively. We then define the distance between *G*^*S*^ and *G*^*T*^ in Equation 2 as the Frobenius norm of the difference between their respective 3D adjacency tensors, as shown in Figure 1.


$$\begin{array}{l} D\left(G^{S},G^{T}\right)=\sqrt{\underset{\left(i,j\right)\in \Omega }{\sum }\|E_{ij}^{S}-E_{ij}^{T}{\|}_{2}^{2}} \end{array}$$ (2)

This formulation targets humanoid-like bodies represented by a shared landmark set and assumes a known semantic correspondence between source and target landmarks. In our experiments, this correspondence is specified manually by assigning each graph vertex to the same anatomical role on the humanoid target. Topology changes such as extra limbs or unmatched fingers would require additional rules to merge, drop, or redefine landmarks.

### 3.3 Proximity weighting on edges

Direct formulation of graph distance on normalized edges presents two significant limitations. First, the rest-pose distances in Equation 1 appear in the denominator of each normalized edge and therefore introduce a static edge-dependent scaling in the unweighted graph distance. For instance, in T-pose-normalized graphs, the foot-to-foot rest-pose distance is much smaller than the wrist-to-wrist distance, so the same absolute displacement produces a larger normalized change for the foot edge than for the wrist edge. Second, this rest-pose normalization remains fixed once the rest pose is chosen and therefore cannot reflect motion-dependent changes in proximity. Prior research (Basset et al., 2022) underscores the importance of proximal relationships between body parts in human motion perception, suggesting that shorter edges in motion graphs warrant greater weighting. Static rest-pose normalization alone therefore cannot capture dynamically evolving proximal relationships across motions.

To address these limitations, we introduce proximity-based weighting to the normalized graph. Utilizing softmax for proximity weighting, we derive a proximity-weighted graph distance metric $D_{w}\left(G^{S},G^{T}\right)$ as Equations 3–5:


$$\begin{array}{l} D_{w}\left(G^{S},G^{T}\right)=\sqrt{\underset{\left(i,j\right)\in \Omega }{\sum }\sigma \left(d_{ij}^{S};c\right)\cdot \|E_{ij}^{S}-E_{ij}^{T}{\|}_{2}^{2}} \end{array}$$ (3)

$$\begin{array}{l} d_{ij}^{S}=\|p_{j}^{S}-p_{i}^{S}\| \end{array}$$ (4)

$$\begin{array}{l} \sigma \left(d_{ij}^{S};c\right)=\frac{\exp\left(-c\cdot d_{ij}^{S}\right)}{\sum_{\left(m,n\right)\in \Omega }\exp\left(-c\cdot d_{mn}^{S}\right)} \end{array}$$ (5)

in which $d_{ij}^{S}$ is the distance between vertex *i* and vertex *j* in the current source motion frame, and *c* is a scaling factor that modulates the importance of proximity, where larger values denote greater emphasis on proximity. Accordingly, the softmax weights vary over time with the source motion and emphasize body parts that are currently proximal.

### 3.4 Graph distance for motion retargeting

Within this framework, motion retargeting is formulated by optimizing the target joint angles and root height to minimize the proximity-weighted graph distance between the resulting target motion graph and the source motion graph.

**Figure 2:**
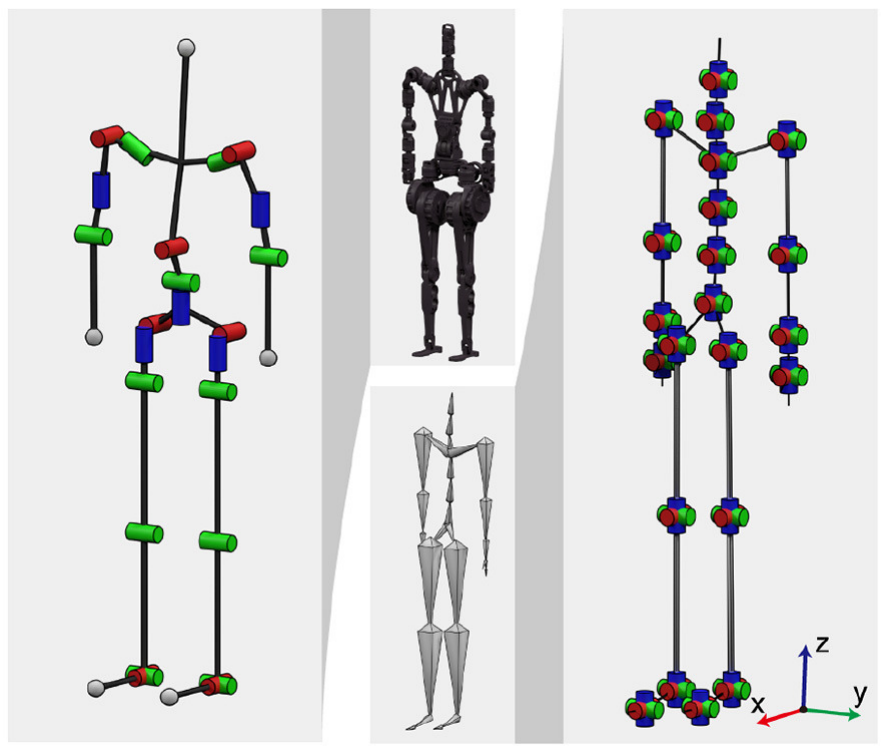
DoFs definition of GR-1 **(Left)** and CMU motion capture data **(Right)**. The GR-1 has substantially fewer DoFs than the CMU mocap skeleton. The shoulder pitch joints of GR-1 (the green axes around shoulder) are oriented at approximately 20 degrees to the horizontal plane, indicating joint configuration differences. For clarity, the finger joints of the CMU mocap skeleton are omitted.

To demonstrate the applicability of this formulation, we consider motion retargeting from mocap data to the GR-1 robot as a representative case study, addressing significant structural divergences, including disparities in DoF configurations and skeletal morphologies. Excluding the root DoFs, the GR-1 robot has a total of 23 rotational DoFs ***q*** ∈ IR^23^, while the CMU mocap data use a total of 90 DoFs to describe the motion. The positions and rotational axes of these DoFs are illustrated in Figure 2. The joint angles of GR-1 are constrained to [***q*_*l*_**, ***q*_*h*_**].

We define 14 vertices, including the wrists, elbows, shoulders, neck, pelvis, knees, ankles, and toes, on both the source mocap skeleton and target GR-1 robot (Figure 1). Their positions w.r.t. the root frame are represented as ***p*^*S*^** ∈ IR^14 × 3^ and ***p*^*T*^** ∈ IR^14 × 3^. Given joint angle configurations, ***p*^*T*^** can be derived through forward kinematics as Equation 6.


$$\begin{array}{l} p^{T}=FK\left(q\right) \end{array}$$ (6)

Edges between ground vertex and other vertices are denoted as *E*_*ig*_, which are represented by the normal vectors from the vertices to the ground plane in the world coordinate system.

We assume that the source mocap skeleton and the target humanoid share a pelvis/root body with equivalent semantic meaning. Under this assumption, the robot root orientation is directly transferred from the source mocap data without additional normalization. This assumption is less suitable when the target uses a different root location, or when the source and target use different axis conventions or reference posture frames, in which case an additional root frame transformation is required. Horizontal translation and root height are handled separately in the formulation. We scale the mocap's horizontal positions by a factor λ to obtain the robot's horizontal position, while introducing the root height *h* as an optimization variable. The scaling factor λ is heuristically defined as the ratio of inter-foot distances in rest poses between mocap data and the robot, encouraging foot-ground kinematic consistency and reducing unnecessary foot-skate artifacts.

Using 14 skeletal vertices and one additional ground vertex, we abstract motion graphs for both the source motion capture data (*G*^*S*^) and target GR-1 robot (*G*^*T*^), formulating the motion retargeting problem as the following constrained optimization problem over the robot joint angles in Equation 7.


$$\begin{array}{ll} \arg\underset{q,h}{\min}\; & D_{w}\left(G^{S},G^{T}\left(q,h\right)\right)\\ \text{s.t. } & q\in \left[q_{l},q_{h}\right] \end{array}$$ (7)

Within this formulation, non-unique solutions can arise when distinct joint configurations induce similar spatial relationships among graph vertices, particularly for weakly constrained DoFs. In implementation, we augment the graph-distance objective with a small joint-space regularization term that biases the joints toward their rest-pose values. This term mainly influences weakly constrained DoFs and thereby mitigates branch switching caused by insufficient kinematic constraints.

Platform-specific DoFs without direct counterparts in the source motion still enter the graph-distance objective through the forward kinematics from ***q*** to *G*^*T*^. They are therefore included in ***q*** and solved jointly with the other target DoFs by minimizing this objective under joint-limit constraints. When these DoFs affect the graph-distance term only weakly, the same regularization favors stable, rest-pose-biased configurations.

Derived through forward kinematics, *G*^*T*^ contains highly non-linear terms. This characteristic can cause weighted graph distance optimization to converge to local optima when using gradient-based methods like L-BFGS-B, especially for contact-rich motions. We therefore employ the differential evolution algorithm (Storn and Price, 1997) as an alternative solver. To improve initialization and temporal continuity, we solve the first frame using differential evolution and optimize subsequent frames with L-BFGS-B initialized from the previous frame's solution. A detailed comparison between L-BFGS-B, the differential evolution solver, and the hybrid strategy is provided in Section 1 of Data Sheet 1 in Supplementary material.

With the joint-space regularization and the hybrid initialization strategy, we only rarely observe branch switching caused by unresolved solution ambiguity in practice.

## 4 Results

### 4.1 Retargeting for diverse motions and humanoids

**Figure 3:**
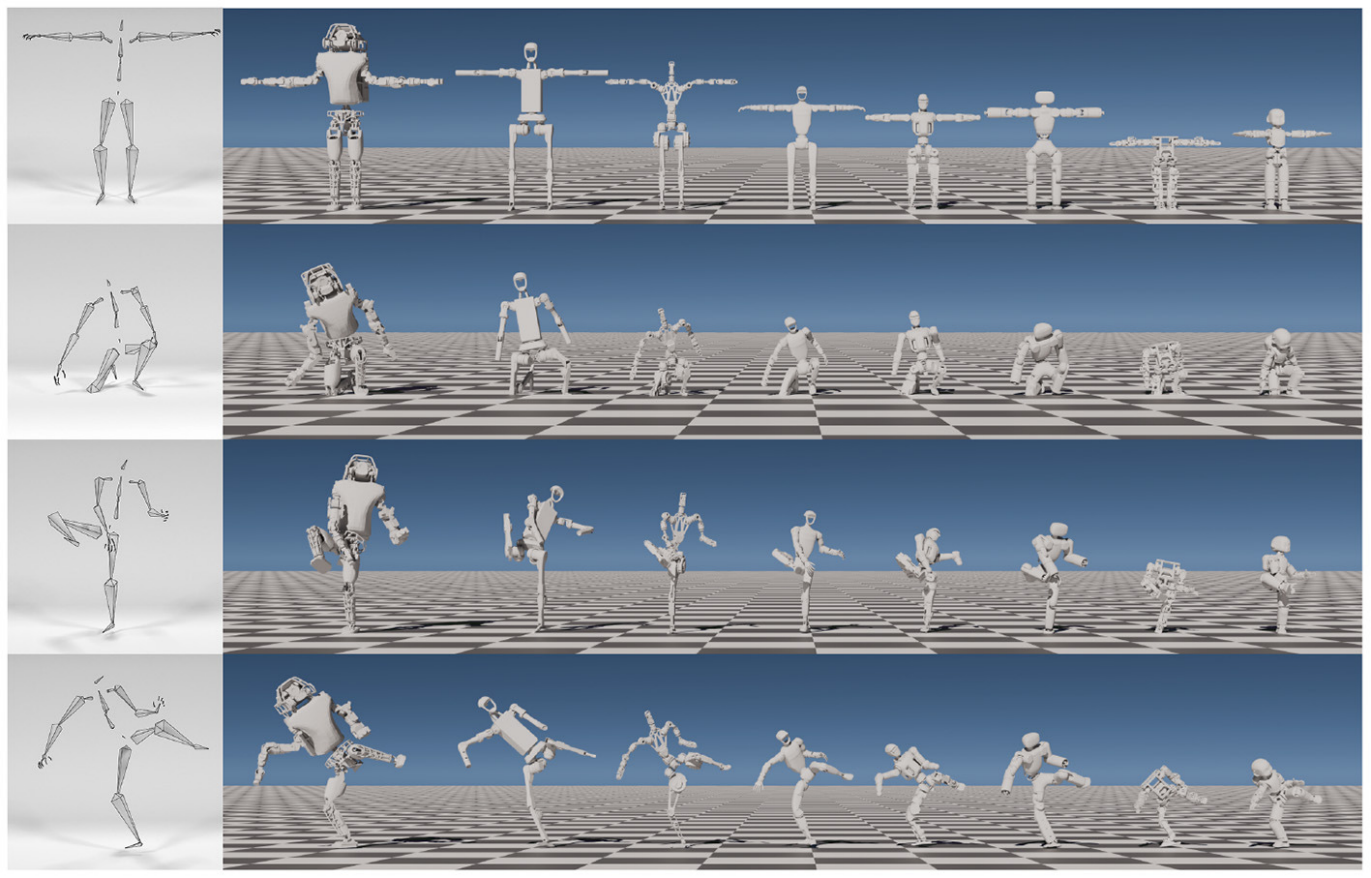
Retargeting mocap data to eight robots. The left column shows the original/reference mocap poses. The retargeted robots are, from left to right after the reference column: (1) Atlas from Boston Dynamics, (2) H1 from Unitree, (3) GR-1 from Fourier Robotics, (4) G1 from Unitree, (5) N1 from Fourier Robotics, (6) X1 from Agibot, (7) Berkeley Humanoid Lite, and (8) iCub from Italian Institute of Technology.

To demonstrate the versatility of our proposed motion retargeting method based on optimization, we retarget diverse motions to eight humanoid robots (see Figure 3) whose models are available online.

Notably, these robots differ in size, body proportions, and DoF configurations, as shown in Table 1. We designate waist DoFs as r (roll), p (pitch), and y (yaw). Limb DoFs (legs and arms) are expressed as *A* + *B*, where *A* represents core DoFs common across platforms (hip roll/yaw/pitch and knee for legs; shoulder roll/yaw/pitch and elbow for arms), while *B* denotes platform-specific unique DoFs that are expressed using the same rotational notation (r/p/y) as the waist DoFs. In terms of size, iCub matches the proportions and stature of a typical child, while H1 exhibits adult human size and proportions. In addition, the number of DoFs varies from 19 to 29, and joint configurations also differ. H1, N1, and Berkeley Humanoid Lite lack waist pitch and roll DoFs, limiting their expressiveness. These robots also differ in wrist and ankle DoFs, with some lacking ankle roll or wrist yaw joints. Table 1 therefore summarizes the humanoid robots evaluated in this study, which share semantic body parts while differing in DoF configurations and body proportions.

**Table 1:** Robots' variations in size and DoF configurations.

Robots	Sizes	Total DoFs	DoFs		
			Waist	Legs	Arms
Atlas	197 cm	29	3(**rpy**)	4+2(**rp**)	4+3(**pyy**)
H1	178 cm	19	1(**y**)	4+1(**p**)	4
GR-1	165 cm	23	3(**rpy**)	4+2(**rp**)	4
G1	132 cm	23	3(**rpy**)	4+1(**p**)	4
N1	130 cm	23	1(**y**)	4+2(**rp**)	4+1(**y**)
X1	130 cm	23	3(**rpy**)	4+2(**rp**)	4
Humanoid Lite	100 cm	22	0	4+2(**rp**)	4+1(**y**)
iCub	104 cm	23	3(**rpy**)	4+2(**rp**)	4

The bold symbols r, p, and y denote the roll, pitch, and yaw DoFs, respectively, of the waist joint in the Waist column, the ankle joints in the Legs column, and the wrist joints in the Arms column.

**Figure 4:**
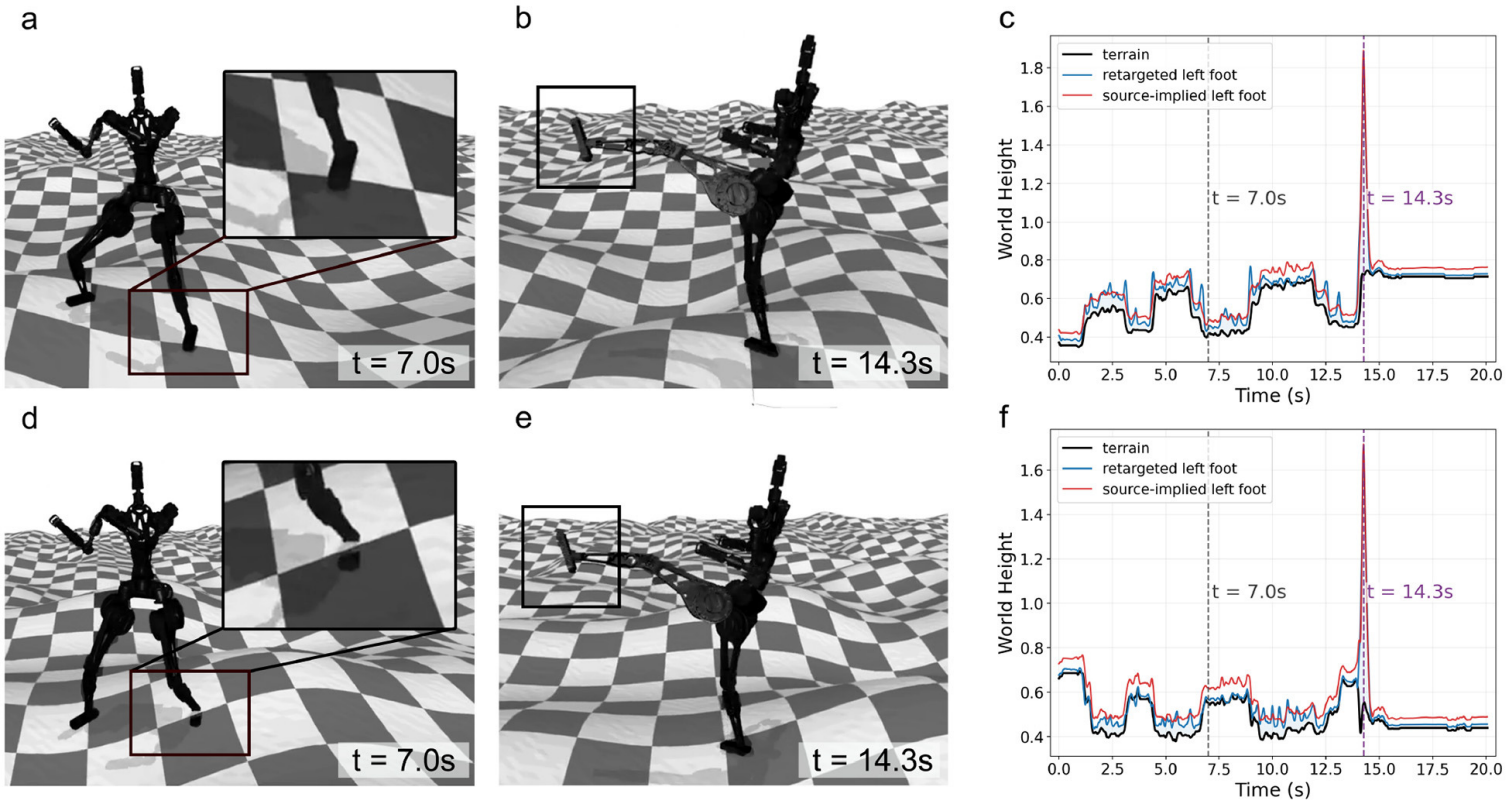
Foot-ground relationship on uneven terrain for the *extended 1* motion. Panels **(a, d)** show the retargeted pose at *t* = 7.0 s on two different height maps, with zoomed views of the left foot. Panels **(b, e)** show the corresponding retargeted pose at *t* = 14.3 s. Panels **(c, f)** plot the terrain height, retargeted left-foot height, and source-implied left-foot height over time. Because the source skeleton and target robot have different global scales, we first rescale the source left-foot clearance (its height above the source ground) to the target robot. The scale factor is the ratio of their T-pose pelvis-to-ground heights. We then add the scaled clearance to the local terrain height to obtain the source-implied left-foot height. The retargeted left-foot trajectory remains close to the source-implied trajectory while following the terrain-height profile, indicating that the foot-ground clearance is approximately preserved across both uneven terrains.

Across these different robots, our method produces qualitatively plausible retargeting results for motions that include knee-ground and foot-ground proximity (see Supplementary Video 1 for animation). Because foot-ground contact is not imposed as a hard optimization constraint, these results should be interpreted as preservation of approximate ground-related spatial relationships rather than as a guarantee of exact contact timing. For foot-ground relationships, the proximity weighting and ground vertex encourage the optimized feet to follow the source-implied clearance relative to the local ground. This behavior is visible in the non-flat-terrain examples of Supplementary Video 2 and in Figure 4, where the gap between the retargeted left-foot trajectory and the terrain remains close to the corresponding source-implied gap across two uneven height maps.

**Figure 5:**
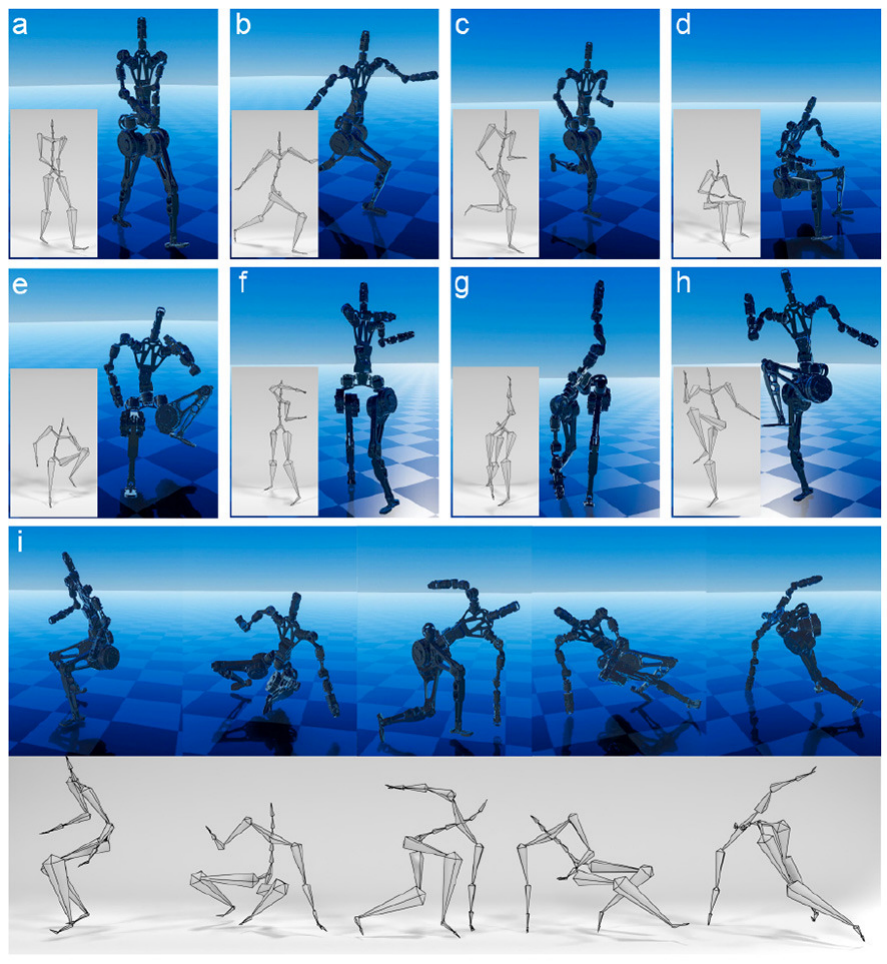
Retargeting CMU mocap database to the GR-1 robot. We selected eight iconic movements **(a–h)** from the database to evaluate spatial relationships and close interactions between body parts and the ground. The lower-left wireframe inset in each of **(a–h)** shows the corresponding original/reference mocap pose. Sequential frames of motion labeled *FancyFootWork* **(i)** illustrate how introducing the ground as a graph vertex encourages ground-related spatial relationships in a motion with multiple low-clearance phases. In **(i)**, the bottom wireframe strip shows the original/reference motion sequence corresponding to the retargeted GR-1 frames shown above.

To further demonstrate the generality of our method for diverse motions, especially those with complex ground interactions and close body interactions, we retarget motions from the CMU mocap database to GR-1, as shown in Figure 5 and Supplementary Video 3.

### 4.2 Spatial relationship preserving

**Figure 6:**
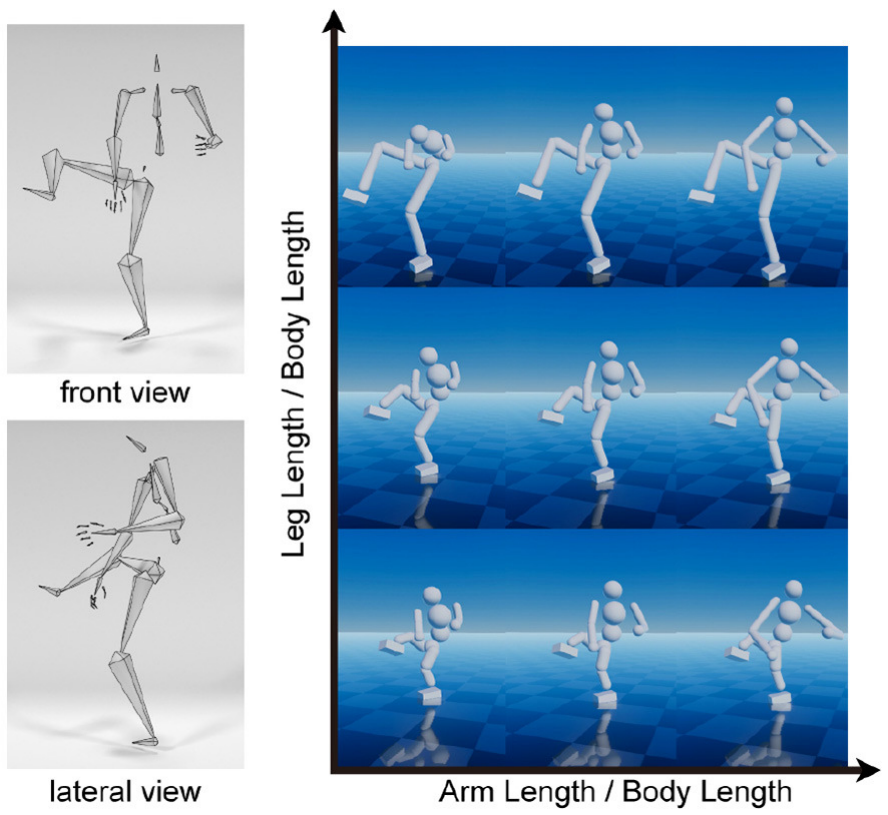
Retargeting results of the Zeibekiko dance [AMASS database (Mahmood et al., 2019)] to the parameterized robots. The left panel displays the original mocap data, while the right panel presents retargeted motions across robots with proportional variations. As arm length increases progressively from left to right, compensatory kinematic adaptations emerge: elbow flexion angles systematically increase while torso flexion decreases. These adjustments preserve critical spatial relationships among the wrist, hip, and knee joints across the robots.

Prior human biological motion perception studies (Tang et al., 2008; Basset et al., 2022; Harada et al., 2004) indicate that perceived motion similarity depends on pairwise spatial relationships between body parts, expressed in terms of relative distances and inter-landmark directions. Based on this perceptual interpretation, we define spatial-relationship preservation as retaining the relative distances and directions between semantic body landmarks before and after retargeting. To evaluate this preservation during motion retargeting, we conducted systematic tests across robots with varying body dimensions and proportions. Specifically, we parameterized robot body proportions using arm-to-body and leg-to-body ratios. We generated 441 distinct robot configurations, and each configuration was retargeted to an identical pose to assess spatial consistency. The source motion and the optimized poses of these proportionally varied robots are illustrated in Figure 6. We also provide animations of retargeted motions on these robots for visual comparison in Supplementary Video 4. This analysis therefore evaluates sensitivity to body-proportion changes within a fixed humanoid topology rather than to arbitrary topology changes.

When assessing spatial relationship preservation across characters, an ideal metric must be robust to structural variations. Directly comparing relative distances and inter-landmark directions does not satisfy this requirement, as absolute distances change with body size, and directional vectors can also shift under proportional differences. We therefore use distance ordinal consistency as a morphology-tolerant proxy for evaluating the distance component of spatial-relationship preservation. This metric relies on the ranking hierarchy of inter-joint distances rather than their absolute magnitudes, so preservation is evaluated by whether landmark pairs that are relatively close or far in the source pose remain relatively close or far after retargeting.

To formally quantify this ordinal consistency, we evaluate inter-joint distances before and after retargeting using Spearman's rank correlation coefficient ρ (Spearman, 1904). This metric, previously used by Randall et al. (2023) to measure temporal motion similarity due to its robustness to non-linear transformations and insensitivity to outliers, is here employed to assess the preservation of relative distance structure. Conceptually, the process involves ranking all inter-joint distances in each pose and quantifying the degree of agreement between the two resulting rank sequences. The objective is not to require identical Euclidean distances across morphologies, but to test whether the retargeted pose preserves the source pose's ordinal proximity structure. Mathematically, ρ is defined as the Pearson correlation between the rank variables as Equation 8:


$$\begin{array}{c} \rho =\text{corr}\left(R\left(d^{S}\right),R\left(d^{T}\right)\right) \end{array}$$ (8)

in which *d*^*S*^ and *d*^*T*^ denote arrays of inter-joint distances of the source pose (e.g. mocap data) and target pose (e.g. retargeted robot pose), respectively, and *R*(*d*) indicates the rank array obtained when *d* is sorted.

**Figure 7:**
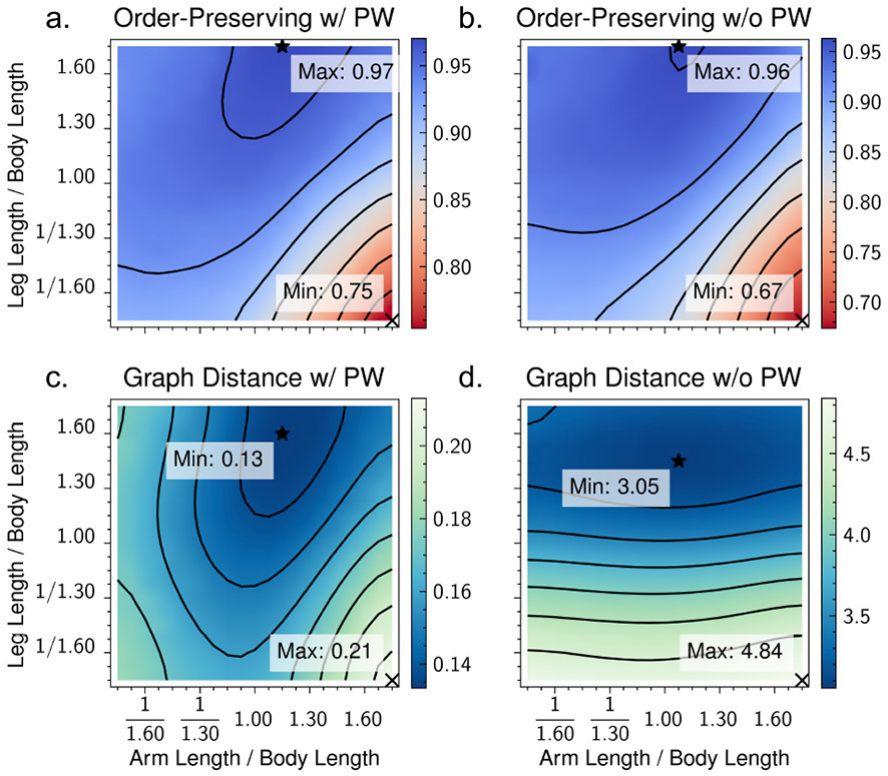
Spatial distance order-preserving consistency and optimized graph-distance metrics before and after retargeting across proportionally varied robots: **(a)** weighted order-preserving consistency, **(b)** unweighted order-preserving consistency, **(c)** optimized weighted graph distance, and **(d)** optimized unweighted graph distance. Weighted and unweighted order-preserving consistency denote the same Spearman-rank metric computed on poses retargeted using the proximity-weighted and unweighted objectives, respectively. Compared with proximity-weighted optimization **(c)**, the unweighted approach **(d)** exhibits reduced order-preserving consistency and attenuated correlation between order-preserving consistency and graph distance.

A Spearman's rank correlation coefficient exceeding 0.7 typically indicates strong positive order-preserving consistency. Figure 7a presents Spearman's rank correlation across our 441 systematically sampled body-proportion configurations, with poses optimized using our method. The results show strong correlations (ρ > 0.7) across a wide range of anthropometric configurations.

The highest order-preserving consistency (star in Figure 7a) and lowest graph distance (star in Figure 7c) are both observed near the original mocap skeleton's proportions (arm-to-body ratio ≈ 1.17, leg-to-body ratio ≈ 1.78), whereas the lowest consistency and the highest graph distance (the cross marks at the right-bottom corner of Figures 7a, c) occur under the same set of extreme proportional configurations. This pattern reveals a strong inverse correlation between the order-preserving consistency and the graph distance metric across proportionally scaled robots. These results indicate that minimizing graph distance helps preserve the spatial relationships encoded by inter-landmark vectors and maintains order-preserving consistency in inter-joint distance rankings. Consequently, graph distance minimization serves as a proportion-robust objective for motion retargeting within this analysis.

Building upon this methodology, we evaluate the significance of proximal weighting for spatial-relationship preservation, with unweighted graph distance *D*(*G*^*S*^, *G*^*T*^) as the optimization objective. The resulting order consistency and unweighted graph-distance metrics are visualized in Figures 7b, d.

As previously described in Section 3.3, the rest-pose normalization in Equation 1 implicitly biases the unweighted graph distance. In the adopted T-pose configuration, the inter-foot distance is smaller than the inter-hand distance, causing variations in leg proportions to have a larger impact on the graph distance. This trend is evidenced in the unweighted graph-distance results of Figure 7d, where changes in leg length (vertical axis) significantly affect graph distance while arm length variations have a minor effect. Comparing the weighted and unweighted order-preserving consistency in Figures 7a, b, we note that the absence of proximity weighting reduces order-preserving consistency, confirming that proximity weighting both helps preserve spatial relationships during retargeting and mitigates bias imposed by the rest pose.

### 4.3 Comparison experiments

We compare our graph-distance-based retargeting method (GD) against four representative methods for humanoid robots: two classical baselines, (1) an inverse kinematics method (IK) and (2) a joint mapping method (JM), and two state-of-the-art optimization-based methods, (3) PHC (Luo et al., 2023) and (4) GMR (Araujo et al., 2025; Ze et al., 2025). The inverse kinematics method calculates joint angles from end-effectors' positions and orientations via inverse kinematics. The joint mapping method directly transfers the source joint angles to the target or derives the target joint angles through simple geometric calculations, with a manually defined mapping between DoFs. Implementation details of the IK and JM baselines are provided in Section 2 of Data Sheet 1 in Supplementary material. For all compared methods, we use the same mocap source data without additional smoothing or normalization. For PHC and GMR, we follow the authors' official implementations. In contrast, the IK and JM baselines require robot-specific design choices such as joint correspondences or target definitions, so we carefully tune them for the GR-1 robot only rather than extending them to all robots. Since there is no single robot that is simultaneously supported by all four baselines, we adopt the following evaluation protocol: (1) we compare the visual quality of retargeted motions on two complementary setups, namely GD, IK, and JM on the GR-1 robot, and GD, PHC, and GMR on Unitree's G1 robot; (2) we then conduct a large-scale quantitative comparison across all available robots (the eight robots described in Section 4.1) using the spatial-relationship-preserving metric introduced in Section 4.2.

#### 4.3.1 Visual quality comparison of retargeting methods

**Figure 8:**
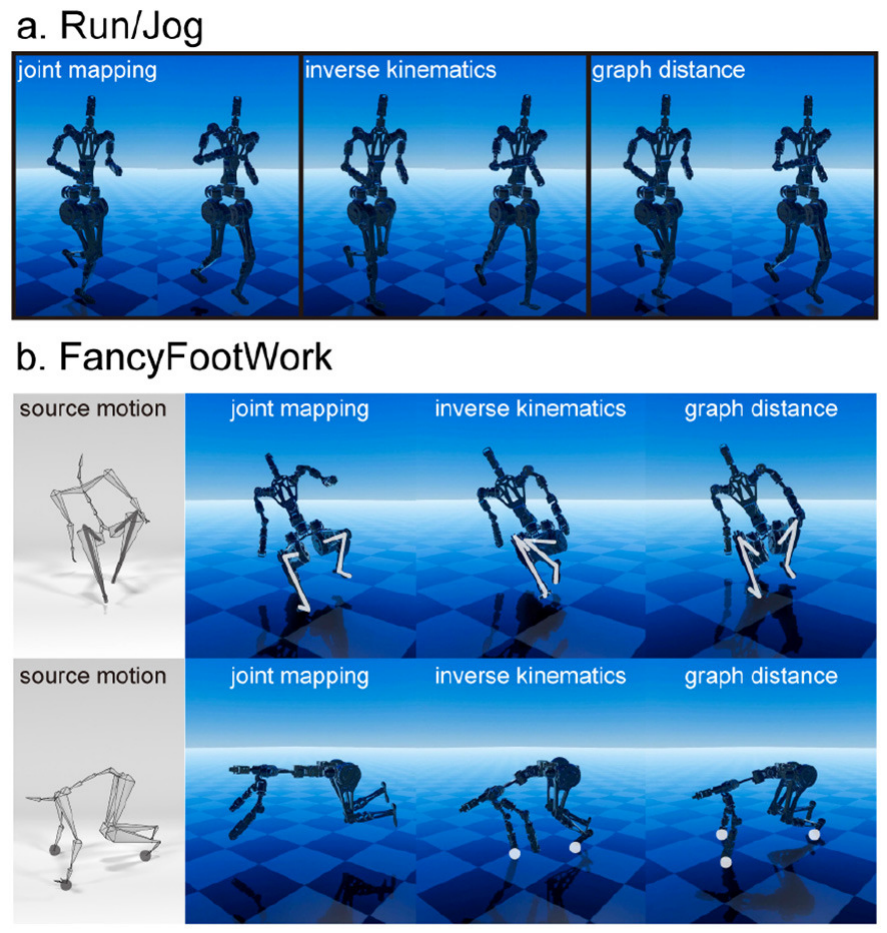
Retargeting results using joint mapping, inverse kinematics, and our method for motion labeled with *Run/Jog* **(a)** and *FancyFootWork* **(b)**. In both panels, method columns from left to right correspond to joint mapping, inverse kinematics, and graph distance. **(a)** shows two representative frames of the simpler locomotion motion *Run/Jog*, for which all three methods appear visually similar. In **(b)**, the leftmost column shows the source motion, and two representative full-body poses are shown for the more contact-rich motion *FancyFootWork*: (1) single-hand contact with legs apart, and (2) quadrupedal ground contact, highlighting method-specific differences. We annotate critical limb segments with solid lines and end effectors in contact with ground with solid dots for *FancyFootWork*.

To qualitatively assess the performance of GD, IK, and JM, we first retarget two trajectories of different complexity from the CMU mocap database, namely *Run/Jog* and *FancyFootWork*, to the GR-1 robot. The resulting motions are shown in Figure 8.

For simple motions such as *Run/Jog* (Figure 8a), all three methods perform comparably, with no significant deviations. However, for more complex motions like *FancyFootWork*, both the IK and JM methods exhibit noticeable artifacts. In the first pose of *FancyFootWork*, both JM and GD accurately capture the leg separation, while IK yields markedly reduced hip abduction compared to the reference (polygonal line in Figure 8b). Although IK preserves the left foot's orientation, it introduces substantial misalignment in the thigh and calf orientation due to the robot's limited DoFs. This issue arises when end-effectors' orientation is enforced despite insufficient kinematic flexibility, leading to pronounced errors in intermediate limb segments.

In the subsequent quadrupedal-contact pose, we mark character-ground contact points with dots in Figure 8b. The IK method keeps two of the three marked end effectors close to the ground, while JM exhibits visible limb floating. In contrast, our GD result keeps all three marked end effectors near the ground in this example. The JM method, which focuses solely on joint angles, does not account for end-effector positions relative to the environment. As a result, when there is a significant discrepancy in body proportions between the source mocap data and the robot skeleton, JM leads to noticeable floating issues or interpenetration, particularly for complex motions.

The state-of-the-art methods GMR and PHC generally produce visually plausible results for most AMASS motions; however, similar artifacts can still occur. GMR first applies tuned non-uniform local scaling to the source mocap and then solves an IK problem with rotation and translation constraints via optimization. Conceptually, it behaves similarly to a classic IK approach, but extends the objectives to match multiple key body segments rather than only the end-effectors. However, it still suffers from incorrect joint orientations, as we observed for the motion *capoera* in the AMASS dataset. PHC first estimates shape parameters that fit the source mocap to the target robot in a standing pose, and then optimizes joint angles based on global joint-translation errors. This shape-fitting step can degrade motion quality when the morphology gap between the source human and the target robot is large. For motions that deviate strongly from a standing configuration, we observe severe ground penetration and base floating. These failure cases are illustrated in Supplementary Video 5.

#### 4.3.2 Comparison of retargeting quality based on spatial relationship preservation

**Figure 9:**
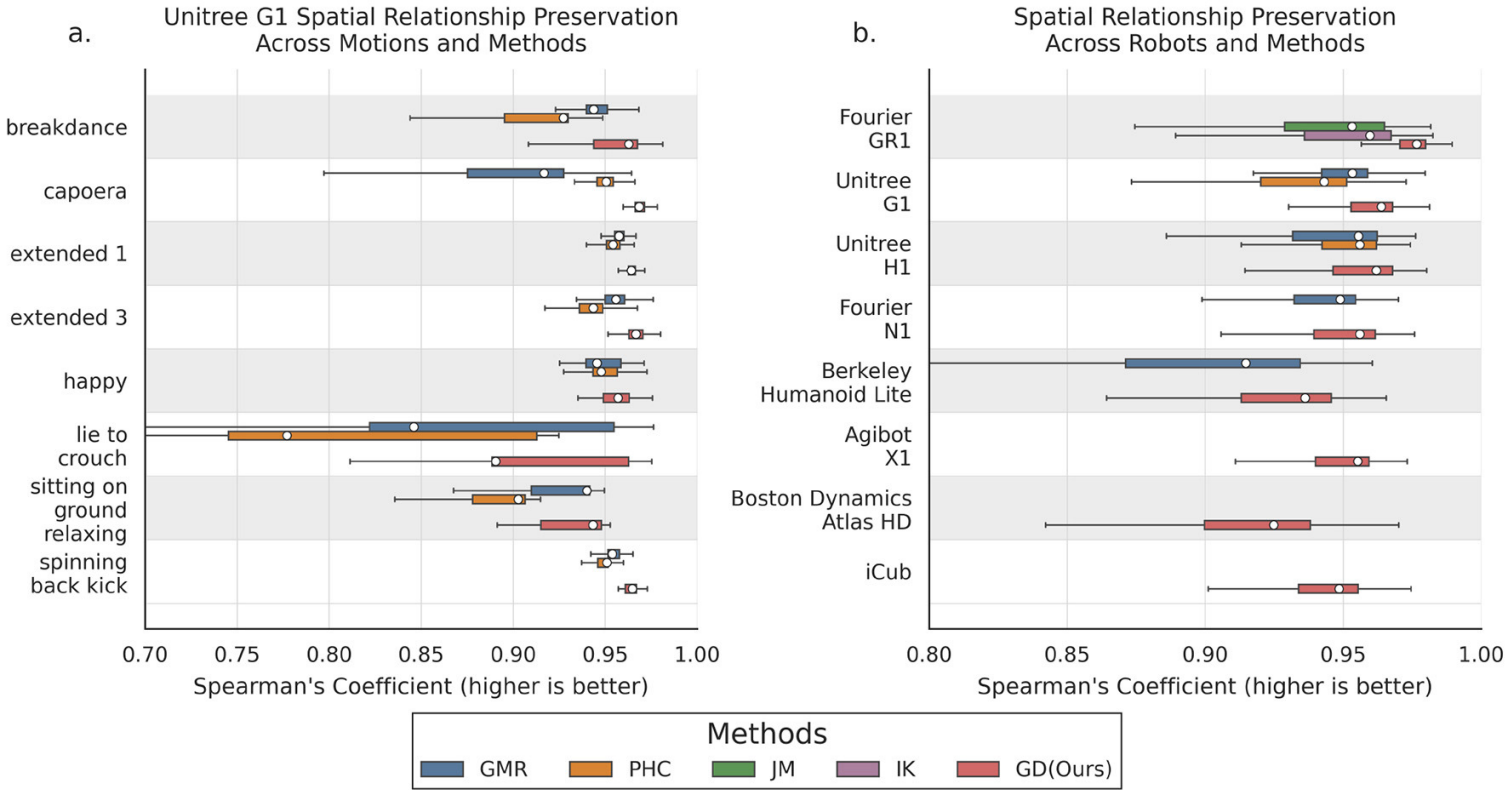
Spatial relationship preservation (SRP) comparisons across motions, robots, and methods. **(a)** SRP distributions on Unitree G1 across the same eight motions used throughout this comparison: *breakdance, capoera, extended 1, extended 3, happy, lie to crouch, sitting on ground relaxing*, and *spinning back kick*, comparing GD, GMR, and PHC; these eight motions are visualized in Supplementary Video 3. **(b)** SRP distributions across robots and methods over the same motion set. We retarget motions (1) to all eight robots using GD, (2) to GR-1 using IK and JM, and (3) to G1 and H1 using GMR and PHC following the official implementations. White dots mark the median SRP values of the corresponding distributions. Higher Spearman-rank correlation indicates better spatial-relationship preservation.

To quantitatively compare the retargeting methods, we evaluate spatial-relationship preservation (SRP) across robots and motions. For each source-target motion pair, SRP is quantified by the Spearman's rank correlation coefficient between the inter-joint-distance rankings of the source motion and those of the corresponding retargeted motion. Operationally, SRP evaluates whether ordinal proximity relations are preserved after retargeting. For example, if the hand is closer to the knee than to the shoulder in the source pose, SRP assigns a higher value to a retargeted pose that preserves this ordering, even though the exact hand–knee and hand–shoulder distances may change with robot body proportions. This makes SRP suitable for comparing retargeted motions across morphologies where absolute distances are not directly comparable but relative proximity structure remains meaningful. SRP is most informative for poses involving salient near-contact or self-contact relationships. SRP is less informative when several inter-landmark distances are nearly equal, because small geometric changes can alter their rank ordering without indicating a clear perceptual difference. For such crowded near-contact configurations, SRP should be interpreted together with visual inspection and graph-distance analysis. We compute SRP for all eight robots described in Section 4.1. We use the same eight motions shown in Figure 9a: *breakdance, capoera, extended 1, extended 3, happy, lie to crouch, sitting on ground relaxing*, and *spinning back kick*. These motions cover both close interactions/contacts and highly dynamic actions.

We illustrate the behavior of SRP as a perceptually motivated evaluation metric using the G1 robot as an example in Figure 9a. For some motions, such as *extended 1, extended 3, happy*, and *spinning back kick*, all methods achieve similarly high SRP values, and the retargeted motions exhibit comparable visual quality. In contrast, for contact-rich motions, individual methods may fail to maintain similarity: PHC performs poorly on *breakdance, lie to crouch*, and *sitting on ground relaxing*, while GMR fails on *capoera*. We show side-to-side comparisons and representative failure cases in Supplementary Video 5. These results support the usefulness of SRP-based Spearman correlation as a motion-similarity metric for evaluating spatial-relationship preservation.

For a broader comparison, Figure 9b further evaluates SRP across all robots and against all four baselines. Overall, GD achieves higher SRP values than the baselines over the set of compared robots and motions, indicating better preservation of spatial relationships. We also observe comparable or more concentrated SRP distributions for most motions, suggesting that GD yields better temporal stability across frames.

### 4.4 Motion-dependent joint sensitivity

An advantage of our proximity-weighted graph distance metric is its ability to adjust the relative importance of different joints based on motion characteristics. Joints along kinematic chains whose endpoints are spatially close exhibit amplified influences on the metric. This behavior is consistent with prior findings that self-contact events are perceptually important in motion.

**Figure 10:**
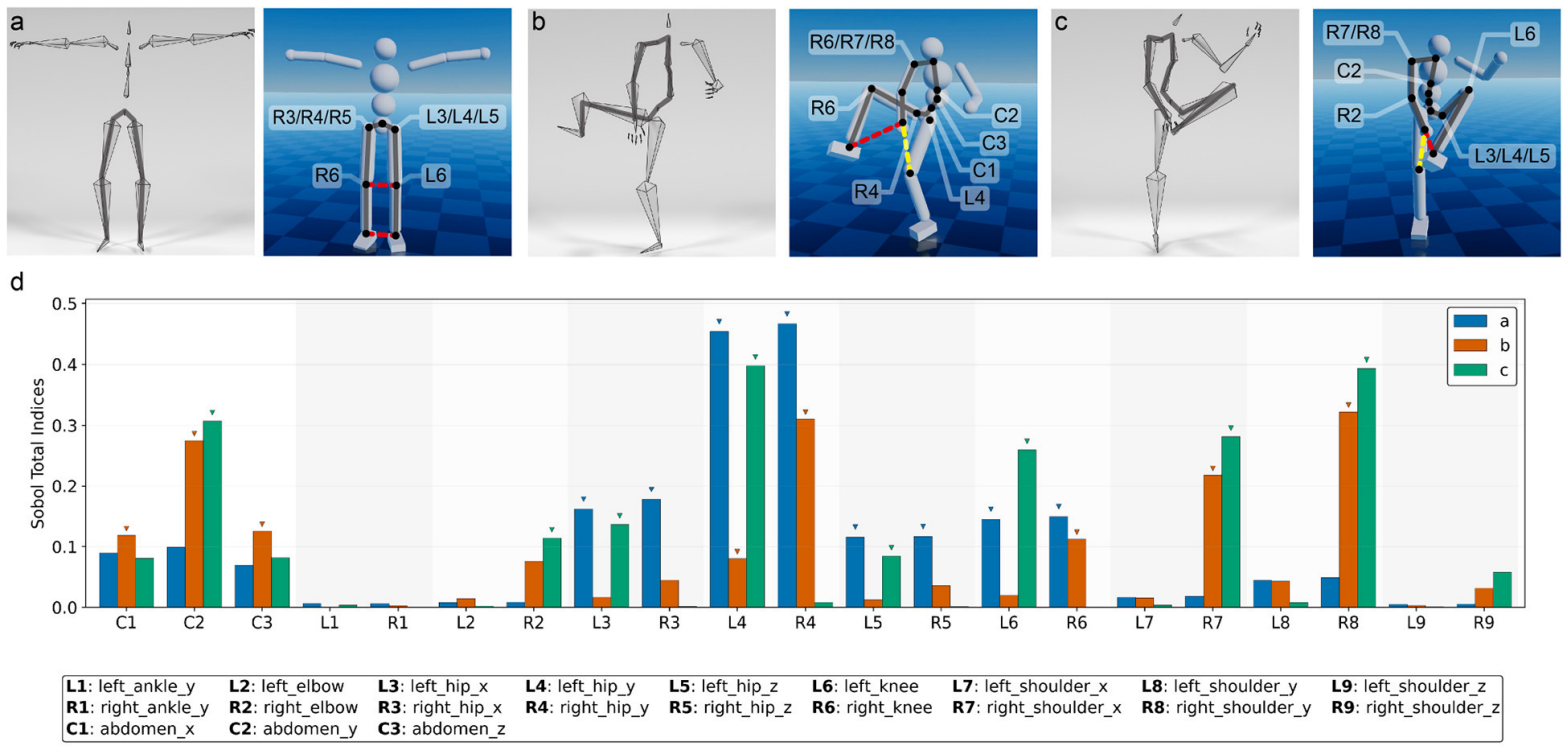
Sensitivity analysis is performed for three representative poses sampled from the Zeibekiko dance shown in Figure 6, selected to exhibit distinct proximal relationships. **(a–c)**: source and retargeted poses, where red dashed lines mark primary proximal relationships along corresponding gray solid kinematic chains, and yellow dashed lines indicate additional proximal relationships observed in the second and third poses. **(d)**: grouped bar chart of Sobol total indices for all 21 evaluated joints across the three poses. Corresponding left and right joints are placed adjacently to support bilateral comparison, and downward triangles on the bars mark the top eight most influential joints for each pose. Joint abbreviations are listed below the chart.

We use Sobol global sensitivity analysis (Sobol, 2001) to examine which joints most strongly influence the proposed proximity-weighted graph distance. Sensitivity analysis is applied to the proximity-weighted graph distance formulation using three representative poses sampled from the Zeibekiko dance shown in Figure 6, chosen to demonstrate varying proximal relationships. The resulting Sobol sensitivity total indices are illustrated in Figure 10 to demonstrate the relative importance of individual joints. Ground vertices are excluded from sensitivity analysis to prioritize inter-body-parts relationships.

We analyzed three different proximal configurations, which respectively demonstrate (1) a symmetric pose with proximity between the legs, (2) proximity between same-side limbs (right wrist and right ankle), and (3) proximity between cross-body limbs (right wrist and left ankle). The first pose exhibits symmetrical left-right joint sensitivity, visible from the adjacent left-right joint bars in Figure 10d and consistent with its inherent bilateral symmetry. The proximal relationship between the legs localizes the top eight graph-distance-sensitive joints, marked by downward triangles in Figure 10d, along the foot-to-foot kinematic chain via the hips and knees. Similarly, in both the same-side limb-proximity and cross-body limb-proximity configurations, localized joint sensitivity patterns emerge along their respective limb-to-limb kinematic chains. This consistent pattern indicates that our graph distance formulation successfully captures the distinct effects of joints on diverse proximity relationships.

While these consistent patterns validate the core formulation, we noted an exception for the second pose. We found that in the second pose, the left hip joint (*L4*) ranks eighth in graph-distance sensitivity despite lying outside the primary proximity kinematic chain. This phenomenon arises from a mere 17% difference between the primary same-side wrist-ankle distance (red dashed line in Figure 10b) and the cross-body wrist-knee distance (yellow dashed line in Figure 10b), creating a secondary proximity that amplifies the corresponding sensitivities. In contrast, in the third pose, a 62% difference between the cross-body wrist-to-knee distance (yellow dashed line in Figure 10c) and the wrist-ankle distance (red dashed line in Figure 10c) results in less significant *R4* joint influence than the *L4* joint in the second pose, accounting for its absence in the third pose despite the overall similarity between the second and third poses.

## 5 Discussion

We implement motion retargeting as the optimization of the proximity-weighted graph distance. Through motion retargeting experiments across diverse robots and motions, we demonstrate the versatility of our method and its capability to preserve spatial relationships. To rigorously evaluate this preservation, we develop a correlation-based assessment method that is robust to non-linear transformations. Comparative analysis shows that proximity weighting is essential for preserving spatial relationships. More broadly, the proposed graph-based formulation provides a common method for representing, transferring, and assessing motion in embodied humanoid systems.

Classical joint-mapping and inverse-kinematics approaches typically formulate retargeting through joint correspondences, end-effector targets, or task constraints. These formulations are effective in many geometric transfer settings, but contact- and proximity-rich motions often require relational cues because their semantics depend on hand-to-body, limb-to-limb, or foot-to-ground relationships. Relative to recent methods such as GMR and PHC, a practical advantage of the proposed formulation is that it avoids manual local or global skeleton scaling by directly optimizing the target robot's joint angles on normalized, proximity-weighted semantic landmark graphs. Topology-based and interaction-preserving methods such as TMG and OmniRetarget already highlight the value of preserving relational structure in motion retargeting. This study addresses retargeting human mocap to humanoid robots with shared semantic body parts, as reflected by our problem formulation and experiments. Topology changes such as extra limbs or unmatched fingers would require additional manual rules to merge, drop, or redefine landmarks. Because the present study does not include a dedicated user study, we present the graph distance and SRP metrics as perceptually motivated tools rather than as directly validated models of human similarity judgments. A human-subject evaluation remains future work.

Section 3 of Data Sheet 1 reports a supplementary imitation-learning experiment in which graph distance is used as the tracking reward and achieves tracking performance comparable to a conventional Cartesian key-body tracking reward. The experiment provides supplementary evidence that the graph-distance metric can serve as an alternative tracking signal, with a tracking objective that directly encodes inter-landmark spatial relationships. Nevertheless, this downstream result does not remove an important limitation: the retargeting procedure itself is purely kinematic and therefore does not guarantee that the resulting retargeted reference is dynamically feasible or accurately trackable under constraints such as actuation/torque limits, contact feasibility, or the robot's controllable subspace, which is a common challenge for under-actuated systems such as humanoid robots.

The retargeting formulation can also fail in poorly conditioned or ambiguous cases. For humanoids with extreme morphologies, although Figure 7 demonstrates robustness to moderate variations in body proportions, we observe a relatively low spatial-relationship preservation score for some highly non-proportional targets. We also find that our method may struggle when the target robot admits ambiguous joint-space solutions for similar keypoint configurations. This issue is particularly severe near singular configurations (e.g. near full elbow extension), where small perturbations can lead to large changes in the inferred joint angles and may cause discontinuities or branch switching across frames. As we do not directly constrain joint orientations from mocap as part of the objective, we cannot fully eliminate this failure mode. Adding additional keypoints or orientation cues may alleviate ambiguity, but it also increases the number of constraints and can make the optimization more difficult to converge.

Beyond these retargeting-stage failure cases, the proposed graph-based similarity metric also has computational and temporal limitations. High computational complexity from dense edge connections and forward kinematics prevents real-time optimization, and frame-wise processing lacks temporal coherence, requiring post-filters.

## 6 Conclusion

In this work, we adapt an inter-joint graph representation to humanoid motion retargeting by introducing an explicit ground vertex and defining a proximity-weighted graph distance as a quantitative measure of motion similarity, motivated by findings from human motion-perception studies. This similarity metric uses a shared semantic landmark set for humanoid-like skeletons and prioritizes spatial, especially proximal, relationships between body parts and the environment, with minimal manual intervention and tuning.

Future directions include task-specific edge pruning [e.g., the interaction-chain method of Li et al. (2025)] to reduce complexity and local optima, or reformulating optimization as quadratic programming using kinematic constraints for efficiency, similar to the one used by Kim et al. (2021). Temporal coherence could be addressed through time-variant motion graphs with inter-frame dependencies or incorporating dynamic constraints like momentum conservation.

## Data Availability

The source code, example retargeted motion files, and corresponding visualization scripts presented in this study are publicly available in the project repository: https://github.com/chaojie-fu/MAHR.
