# Supplementary material for: Morphology-agnostic humanoid retargeting via perception-motivated graph similarity
Source: Front Neurorobot. 2026 Jul 23;20:1814977. doi: 10.3389/fnbot.2026.1814977 (PMC13442438; doi:10.3389/fnbot.2026.1814977)
Supplement: Supplementary file 1 [file Data_Sheet_1.pdf]

Supplementary Material

1 COMPARISON BETWEEN L-BFGS-B AND DIFFERENTIAL EVOLUTION SOLVERS

We compare the performance of L-BFGS-B and Differential Evolution (DE) solvers on our optimization problem, using implementations from scipy (Virtanen et al., 2020) out-of-the-box. The optimization problem is formulated as minimizing the proximity-weighted graph distance between mocap data from AMASS (Mahmood et al., 2019) and GR-1 robot.

We compared seven solver configurations (one for L-BFGS-B, six DE settings with different maximum iterations) across eight heterogeneous motions including multi-environmental-contact, dancing and material arts. For each individual motion frame, we treat the minimum optimized value across all seven configurations as the global optimum $d^{\star}$, and define the global optimality gap for a given solver setting as $d - d^{\star}$, where d is the optimized value of that setting. The results, shown in Fig. S1, clearly indicate that L-BFGS-B exhibits significantly larger optimality gaps than DE, confirming that L-BFGS-B is heavily affected by the non-convexity of our graph-distance objective and easily converges to suboptimal local minima.

To further illustrate that motions with strong proximity relationships are harder to optimize, Fig. S2 plots the relationship between the initial objective value of each pose and the corresponding optimized value. In our proximity-weighted setting, short inter-joint edges (i.e., proximal relationships) are emphasized, which yields large objective values even when absolute joint errors are not large. Thus, large initial objective values typically indicate important proximity relationships in the motion. As shown in Fig. S2, L-BFGS-B almost never reaches the global minimum, and DE configurations with small maximum iterations are also more likely to get trapped in local minima in this regime, supporting our claim that proximity-rich motions are more challenging to optimize.

While increasing the maximum number of DE iterations can reliably drive the objective toward the global optimum, Fig. S3 shows that this becomes impractical due to the resulting time-accuracy trade-off. It takes the DE solver tens, hundreds, or even thousands of seconds to converge for a single frame. However, for continuous motion sequences, a simple hybrid strategy proves effective: we apply DE only to the first frame of the sequence, and then use L-BFGS-B to optimize subsequent frames, initialized with the solution of the previous frame. As shown in Fig. S4, L-BFGS-B then almost always converges to the global optimum, while keeping the overall computational cost manageable. This hybrid approach combines the robustness of DE with the efficiency of gradient-based optimization.

2 IMPLEMENTATION DETAILS OF INVERSE KINEMATICS AND JOINT MAPPING METHODS

We formulate our Inverse Kinematics (IK) baselines for humanoid retargeting as a per-kinematic-chain IK problem, where we define separate kinematic chains for each limb and solve for joint angles based on the end-effectors' orientations and positions. We detail the implementation below.

We first define five kinematic chains for the robot: the left/right arms, the left/right legs, and an additional torso chain. All chains share the same base link (the pelvis for GR-1). Because they share the base, the left/right arm chains and the torso chain also share some intermediate joints, such as the waist roll/pitch/yaw. In our implementation, we first solve the torso chain using only orientation objectives,

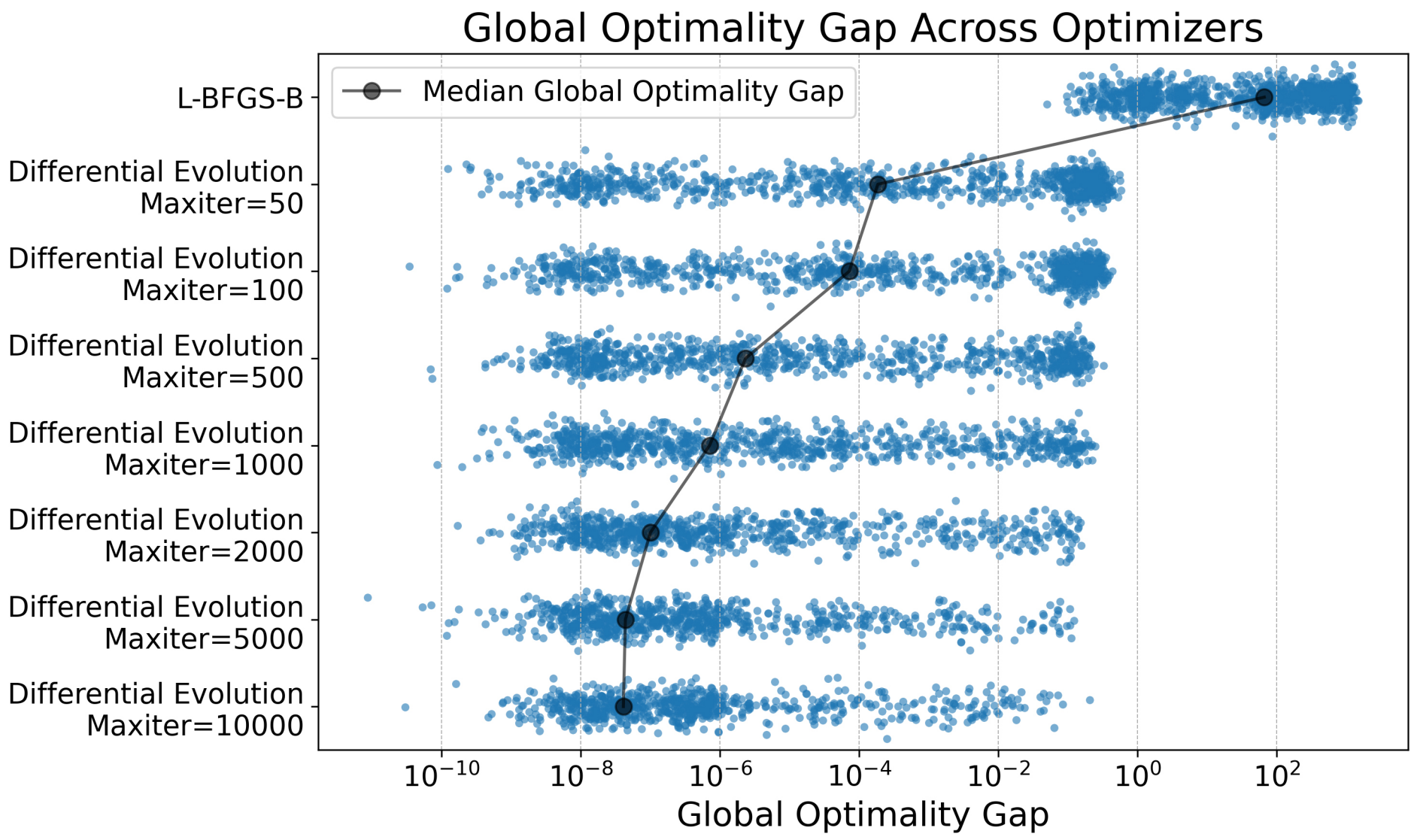

Figure S1. Global optimality gap across solvers including L-BFGS-B and Differential Evolution with varied maximum iterations. L-BFGS-B shows severe local optimum convergence when compared with Differential Evolution. Tendency of convergence is observed as the maximum iteration of differential evolution solver increases.

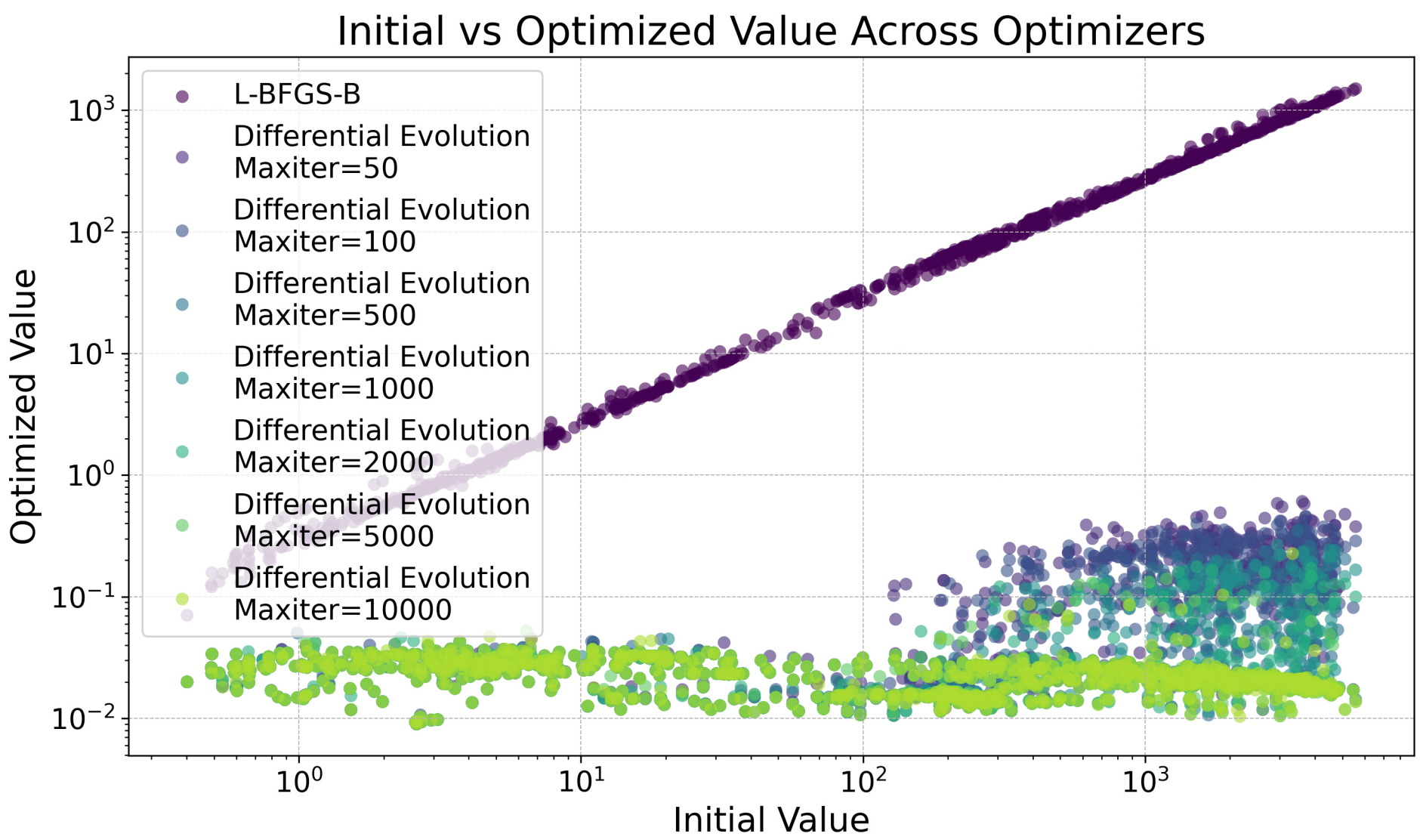

Figure S2. Optimization objective's initial values compared with optimized values. Demonstrating how motions with proximity relationship (high initial value due to proximity weighting) make the optimization objective highly-nonconvex and hard to converge.

obtain the corresponding waist joint angles, and then keep these joint angles fixed when solving IK for the arm chains. For the leg chains, we directly solve all joint angles via IK without sharing joints with the upper body.

For each end-effector, the translational IK target is defined as the end-effector position relative to the base (pelvis) coordinate frame, scaled by a fixed factor. This factor is computed from the source mocap skeleton and the robot dimensions so that, after scaling, the robot and the mocap skeleton share the same

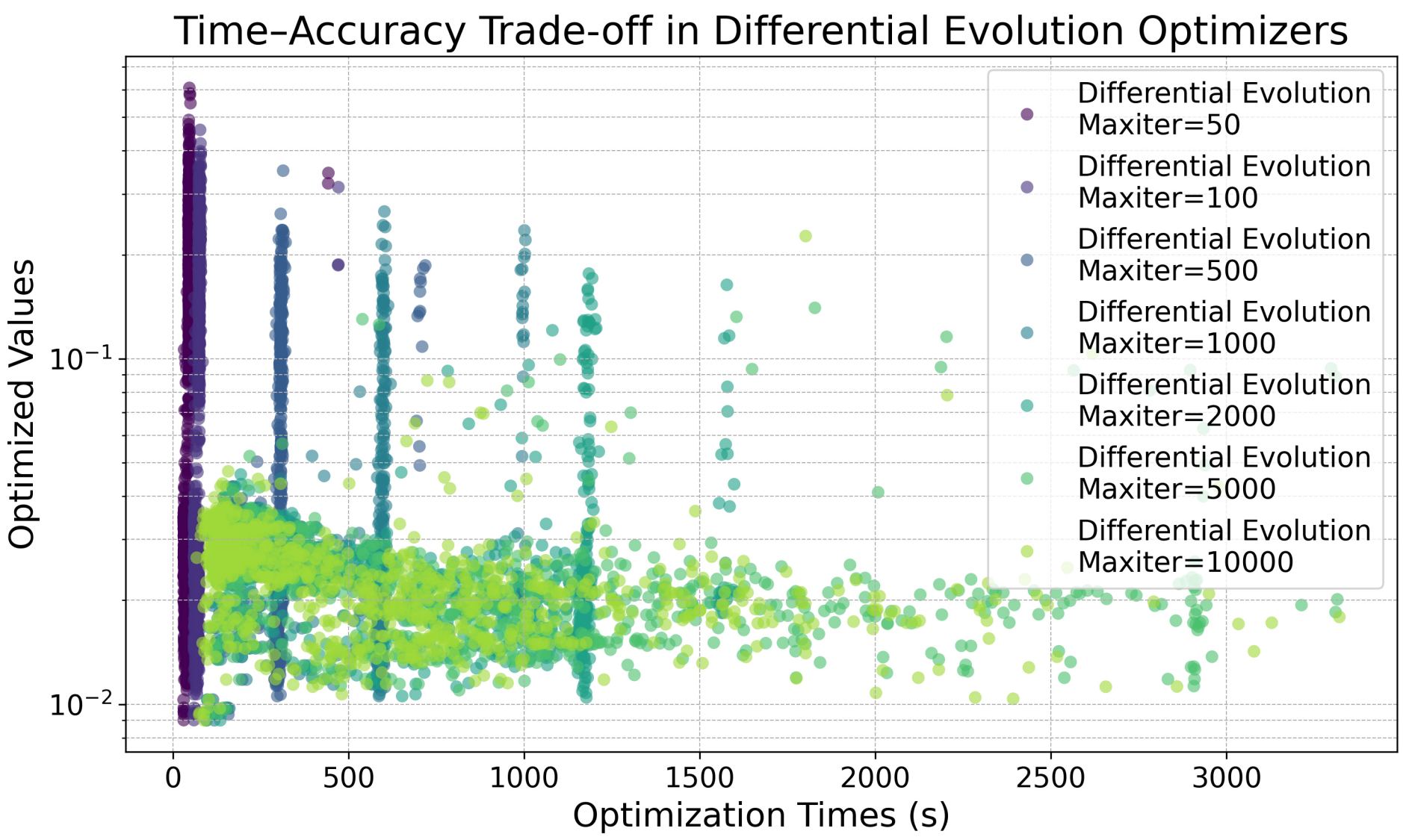

Figure S3. Optimization and accuracy tradeoffs of Differential Evolution. The solution quality relies heavily on the optimization time.

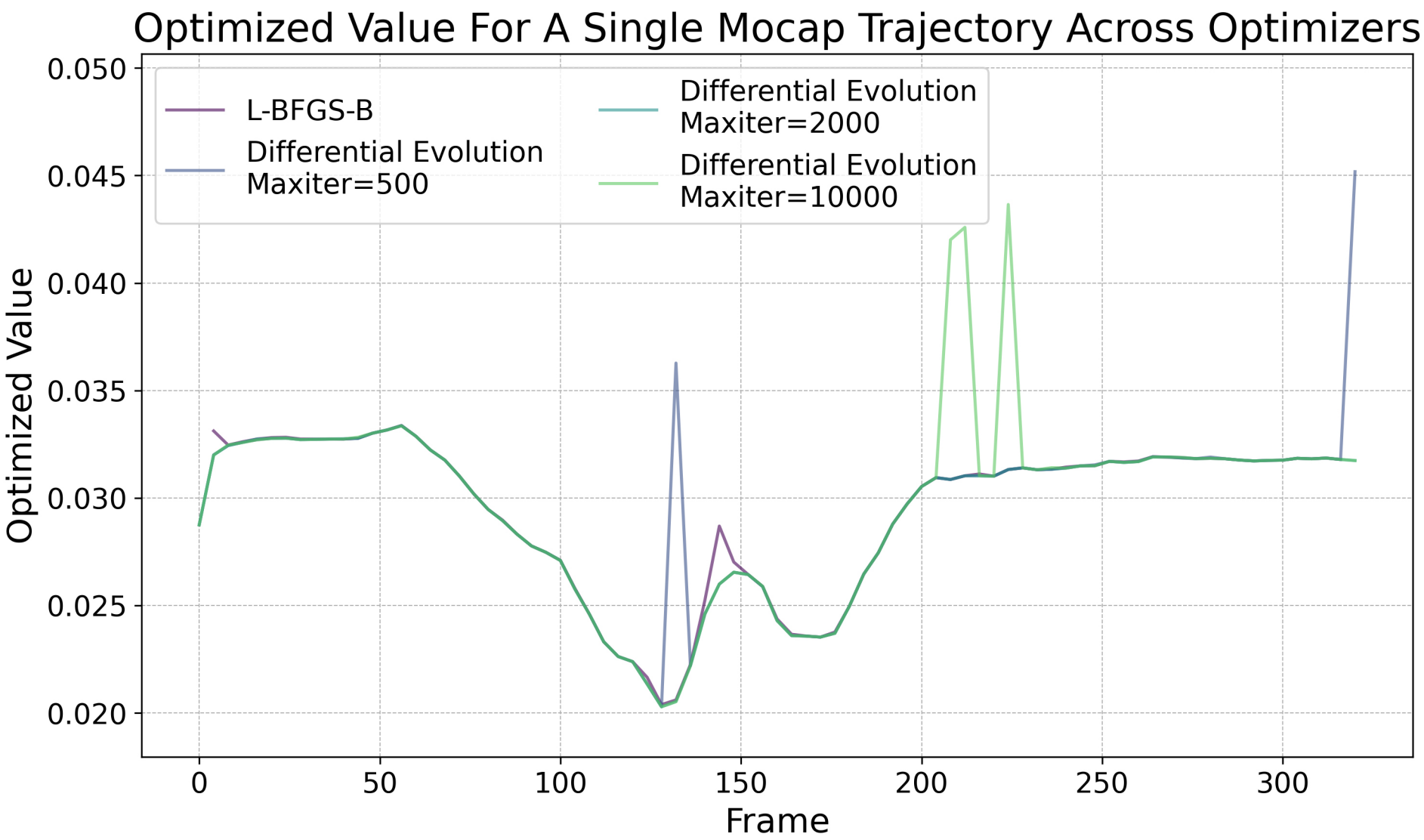

Figure S4. Hybrid strategy for optimizing sequential motion frames achieves near-optimal solution quality, while preserving gradient-based method's efficiency.

standing height. The end-effector orientations are taken from the mocap data and pre-processed so that they represent the same rotation under a common rest pose for both the human mocap skeleton and the robot. Given these per-chain translation and orientation targets, we solve the inverse kinematics using IKPy (Manceron, 2024).

For the joint-mapping (JM) baseline, we largely follow the retargeting pipeline of ASE (Peng et al., 2022). We manually define a mapping from the source mocap joints to the target robot joints, and then compute robot joint angles directly from the mocap joint orientations. This computation is treated as a geometric problem rather than an optimization problem. As an example, consider the waist of the GR-1 robot, whose degrees of freedom follow a roll-pitch-yaw order, corresponding to an intrinsic ZYX Euler representation.

Denoting roll, pitch, and yaw by ϕ, θ, and ψ, respectively, the torso orientation with respect to the pelvis can be written as

$$c_\alpha \coloneqq \cos\alpha \tag{S1}$$

$$s_\alpha \coloneqq \sin\alpha \tag{S2}$$

$$\begin{aligned} R &= R_z(\psi)R_y(\theta)R_x(\phi) \\ &= \begin{bmatrix} c_\psi c_\theta & s_\phi s_\theta c_\psi - s_\psi c_\phi & s_\phi s_\psi + s_\theta c_\phi c_\psi \\ s_\psi c_\theta & s_\phi s_\psi s_\theta + c_\phi c_\psi & -s_\phi c_\psi + s_\psi s_\theta c_\phi \\ -s_\theta & s_\phi c_\theta & c_\phi c_\theta \end{bmatrix} \end{aligned} \tag{S3}$$

From the rotation matrix R, we can recover ϕ, θ, and ψ (assuming no singularity or gimbal lock) as

$$R = \begin{bmatrix} r_{0,0} & r_{0,1} & r_{0,2} \\ r_{1,0} & r_{1,1} & r_{1,2} \\ r_{2,0} & r_{2,1} & r_{2,2} \end{bmatrix} \tag{S4}$$

$$\theta = \arcsin\left(-r_{2,0}\right) \tag{S5}$$

$$\phi = \text{arctan2}\left(r_{2,1}, r_{2,2}\right) \tag{S6}$$

$$\psi = \text{arctan2}\left(r_{1,0}, r_{0,0}\right) \tag{S7}$$

We apply analogous procedures to other three-axis joints such as shoulders and hips, with appropriate modifications to the joint order and axes. For single-axis joints (e.g., knees and elbows), we directly compute the angle between the corresponding bone vectors in the mocap skeleton and assign this angle to the robot joint.

3 SUPPLEMENTARY IMITATION-LEARNING SIDE VALIDATION

This appendix reports a limited reinforcement-learning (RL) side validation of the proposed graph distance in simulation. The purpose is to test whether the same similarity metric used for kinematic retargeting can be inserted as one possible tracking signal in a controlled downstream controller-learning setup. Representative physics-based controller-learning methods also use reference motions as supervisory signals: DeepMimic (Peng et al., 2018) uses imitation and task rewards to learn physics-based controllers from motion clips, and Lee et al. (Lee et al., 2021) show that a single clip can be expanded into a parameterized family of physics-based skills conditioned on variables such as body proportions, task goals, and environmental conditions. We cite these works to situate this experiment as downstream controller-learning context, not as a benchmark against this literature. This experiment is not intended as a benchmark against DeepMimic-style (Peng et al., 2018) or recent humanoid imitation-learning methods, nor as evidence that graph distance is a better reward function for controller learning. We therefore provide the setup, observation and action

spaces, reward terms, and tracking results as supplementary validation rather than as a main contribution of the paper.

3.1 Training Setup

Our training largely follows the DeepMimic framework (Peng et al., 2018), and we use RaiSim (Hwangbo et al., 2018) as the physics simulator. The simulator runs at 400 Hz. Because this experiment is not a deployment study, we omit domain randomization to reduce training variance and accelerate convergence.

To facilitate accurate tracking of highly dynamic motions, we adopt a slightly relaxed actuation setting in simulation by increasing the nominal limits on joint torques and joint velocities relative to the GR-1 hardware specifications. This is mainly motivated by the limited capacity of a few joints (e.g., shoulders) when imitating highly dynamic motions. These choices make the learned policies simulation-only. Real-hardware deployment would require additional system-specific tuning and sim-to-real treatment such as dynamics randomization, and we did not deploy the policies on physical hardware. Importantly, these choices are applied consistently across all reward variants in this supplementary experiment. Therefore, they do not affect the narrow within-study comparison reported below.

The policy is a standard multilayer perceptron (MLP) with two hidden layers of size 256, i.e., 256×256. The policy outputs actions at 100 Hz. We use PPO (Schulman et al., 2017) as the RL algorithm and adopt two common training techniques from DeepMimic, namely Reference State Initialization (RSI) and Early Termination, to improve training efficiency and stability (Peng et al., 2018).

We use a two-stage training scheme: we first train a tracking-focused controller to maximize imitation accuracy, and then fine-tune the policy with additional regularization terms to reduce jerk and contact slips. The reward terms used in the two stages are summarized in Table S2, and the full training hyper-parameters are provided in Table S1.

3.2 Observations and Actions

Our policy acts as a tracking controller. The observation at time t, denoted by $\boldsymbol{o}_t$, consists of proprioceptive observations $\boldsymbol{o}_t^{prop}$ and reference observations $\boldsymbol{o}_t^{ref}$. The proprioceptive part includes joint positions $\boldsymbol{q}_t \in \mathbb{R}^{N_{act}}$, joint velocities $\dot{\boldsymbol{q}}_t \in \mathbb{R}^{N_{act}}$, the gravity direction expressed in the root frame $\boldsymbol{g}_t \in \mathbb{R}^3$, root angular velocity $\boldsymbol{\omega}_t \in \mathbb{R}^3$, root linear velocity $\boldsymbol{v}_t \in \mathbb{R}^3$, and root height $h_t \in \mathbb{R}$.

$$\boldsymbol{o}_t^{prop} = [\boldsymbol{q}_t, \dot{\boldsymbol{q}}_t, \boldsymbol{g}_t, \boldsymbol{\omega}_t, \boldsymbol{v}_t, h_t]$$

The reference observations provide the target state to be tracked and mirror the proprioceptive terms: $\boldsymbol{o}_t^{ref} = \left[\hat{\boldsymbol{q}}_t, \hat{\dot{\boldsymbol{q}}}_t, \hat{\boldsymbol{g}}_t, \hat{\boldsymbol{\omega}}_t, \hat{\boldsymbol{v}}_t, \hat{h}_t\right]$. We include $\boldsymbol{o}_t^{ref}$ so that the policy is conditioned on the specific reference motion. The reference motions are obtained by retargeting AMASS motions using our method and provide frame-wise root positions, root orientations, and joint positions.

The action produced by the policy is first de-normalized and interpreted as target joint positions for PD control. Torques are then computed using a standard PD controller at 400 Hz (simulator rate), where the PD gains are reported in Table S1.

3.3 Reward Function

The reward consists of tracking terms and regularization/penalty terms. We list all reward components and their weights in Table S2. As a within-study diagnostic, we evaluate two alternative tracking rewards: a

Table S1. Hyper-parameters for PPO and PD controller.

Grouped by	Hyper-parameters	Value
PPO	discount factor γ	0.99
	GAE λ	0.95
	value function coefficient	0.5
	entropy coefficient	0.0
	epochs	10
	rollout steps	400
	mini-batches	1
	clip ratio	0.2
	adaptive target KL divergence	0.5
	learning rate	1e-4
PD Controller	Stiffness for hip roll/pitch/yaw	200.0
	Stiffness for knee	200.0
	Stiffness for ankle roll/pitch	60.0
	Stiffness for waist roll/pitch/yaw	60.0
	Stiffness for shoulder roll/pitch/yaw	40.0
	Stiffness for elbow	40.0
	Damping for hip roll/pitch/yaw	5.0
	Damping for knee	5.0
	Damping for ankle roll/pitch	1.5
	Damping for waist roll/pitch/yaw	10.0
	Damping for shoulder roll/pitch/yaw	1.0
	Damping for elbow	1.0
	Maximum joint motor torque	300.0 (Nm)
	Maximum joint motor speed	15.0 (rad/s)

conventional key-body tracking reward and the proposed graph-based similarity reward. Note that each training run uses only one of the two tracking rewards, while the remaining terms are kept identical across runs for a fair comparison. The final reward function is composed of a weighted sum of all items.

Table S2. Reward functions for imitation learning. For tracking rewards such as *Key Body Position Tracking* and *Graph Distance Similarity* (whose weights are marked with $\star$), only one of them is enabled when training.

Reward/Penalty function	Expression	Tracking	Fine Tuning
Root Orientation	$\exp(-12.31 \cdot \|\boldsymbol{g}_t - \hat{\boldsymbol{g}}_t\|^2)$	0.15	0.15
Key Body Position	$\exp(-36.0 \cdot \left\|{}^{fwd}\boldsymbol{p}_t - {}^{fwd}\hat{\boldsymbol{p}}_t\right\|^2 / N_{kb})$	$0.60^\star$	$0.60^\star$
Graph Distance Similarity	$\exp(-9.0 \cdot D_w(G^S, G^T(\boldsymbol{q}_t, h_t))/N_{kb})$	$0.60^\star$	$0.60^\star$
Action Smoothing	$\exp(-60.0 \cdot \|\boldsymbol{a}_t - \boldsymbol{a}_{t-1}\|^2 / N_{act})$	0.05	0.05
Joint Acceleration	$-\|\text{Clip}(\ddot{\boldsymbol{q}}_t, -100, 100)\|^2$	0.00	0.10
Root Linear Acceleration	$-\|\text{Clip}(\boldsymbol{\alpha}_t, -10, 10)\|^2 / 100$	0.00	0.05
Root Angular Acceleration	$-\|\text{Clip}(\boldsymbol{\beta}_t, -10, 10)\|^2 / 100$	0.00	0.05
Contact Slip	$-\max(\boldsymbol{v}_{contact})$	0.00	0.10
Early Termination	-1 if early termination, else 0	1.00	1.00
Alive	1 if alive, else 0	1.00	1.00

3.4 Supplementary Tracking Results

Figure S5 illustrates three skills learned with the graph-distance tracking signal: warmup, boxing, and getting up. Supplementary Video 6 provides visualizations of the corresponding learned policy rollouts. In Figure S5, the ghosted humanoid is the kinematic reference produced by our retargeting method (replayed without physics), while the black humanoid is the physics-simulated policy rollout learned via imitation

learning to track this reference. The overlay illustrates how closely the learned controller follows the retargeted reference over time.

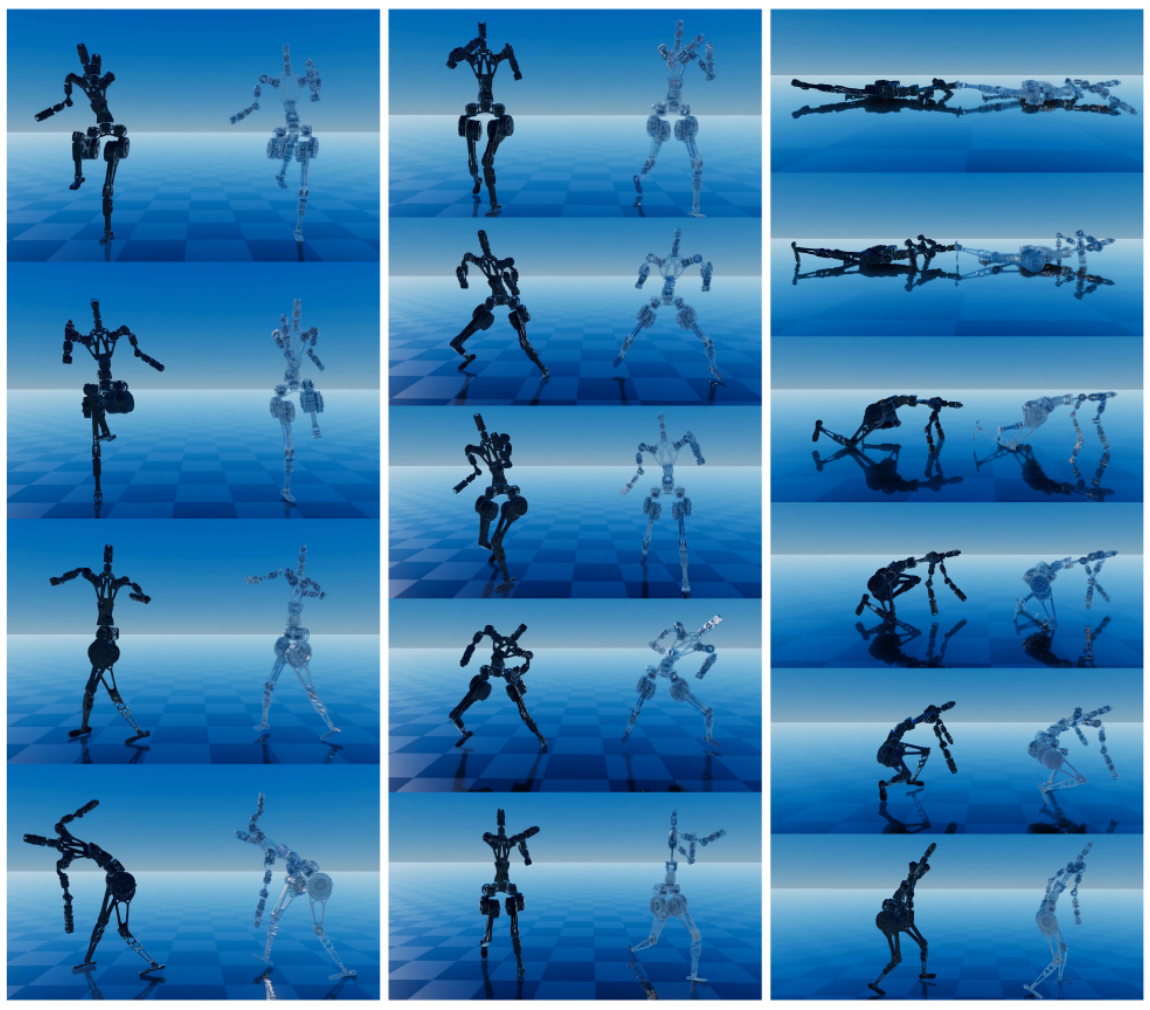

Figure S5. Supplementary imitation-learning side validation for warmup, boxing, and getting up. The black humanoid depicts simulated policy performance, while the ghosted counterpart visualizes reference trajectories.

As a diagnostic comparison within this controlled setup, we report tracking errors obtained with the conventional key-body tracking reward and with the graph-based tracking reward. All training and evaluation settings are kept identical across the two runs, except for the tracking-reward definition. We compare Mean-Per-Joint-Position-Error (defined over Cartesian-position errors of joints w.r.t. the robot root frame ${}^{root}\boldsymbol{p}$) and Mean-Per-Joint-Error (defined over errors of joint angles $\boldsymbol{q}$):

$$\mathrm{MPJPE} = \sum_{t=1}^{T} \left\| {}^{root}\boldsymbol{p}_t - {}^{root}\hat{\boldsymbol{p}}_t \right\| / T$$

$$\mathrm{MPJE} = \sum_{t=1}^{T} \left\| \boldsymbol{q}_t - \hat{\boldsymbol{q}}_t \right\| / T.$$

Table S3. Tracking accuracy in the supplementary side validation. We evaluate Mean Per Joint Position Error (MPJPE) in Cartesian coordinates and Mean Per Joint Error (MPJE) for the motions in Figure S5.

Tracking Error	Motion Warmup (a) $\downarrow$	Motion Boxing (b) $\downarrow$	Motion Get up (c) $\downarrow$
MPJPE (key-body)	$\mathbf{0.0466} \pm 0.0342$	0.0251 ± 0.0190	$\mathbf{0.0225} \pm 0.0182$
MPJPE (graph)	0.0485 ± 0.0367	$\mathbf{0.0248} \pm 0.0158$	0.0291 ± 0.0211
MPJE (key-body)	1.2109 ± 0.5868	0.6537 ± 0.1513	$\mathbf{0.5649} \pm 0.2549$
MPJE (graph)	$\mathbf{0.9695} \pm 0.4871$	$\mathbf{0.5452} \pm 0.1215$	0.6083 ± 0.2078

The results show that the graph-based tracking signal can produce policy rollouts with comparable tracking errors in this limited setting. In this setting, the graph-based tracking signal should be viewed as an alternative tracking signal that emphasizes inter-landmark spatial relationships, rather than as a generally superior reward. We do not interpret these values as evidence that graph distance is a better imitation-learning reward than DeepMimic-style rewards (Peng et al., 2018) or recent humanoid controller-learning methods. MPJPE and MPJE are downstream policy-tracking errors, so they are constrained indicators for comparing reward definitions. Their values are strongly affected by the dynamic feasibility of the retargeted reference and by whether the learned controller can physically track that reference. We therefore use them only as within-study diagnostics rather than as cross-paper evidence for reward superiority.

REFERENCES

Hwangbo, J., Lee, J., and Hutter, M. (2018). Per-Contact Iteration Method for Solving Contact Dynamics. *IEEE Robotics and Automation Letters* 3, 895–902. doi:10.1109/LRA.2018.2792536

Lee, S., Lee, S., Lee, Y., and Lee, J. (2021). Learning a family of motor skills from a single motion clip. *ACM Transactions on Graphics* 40, 1–13. doi:10.1145/3450626.3459774

Mahmood, N., Ghorbani, N., Troje, N. F., Pons-Moll, G., and Black, M. J. (2019). AMASS: Archive of motion capture as surface shapes. In *Proceedings of the IEEE/CVF International Conference on Computer Vision*. 5442–5451

[Dataset] Manceron, P. (2024). IKPy (v3.4.2). doi:10.5281/zenodo.6551105

Peng, X. B., Abbeel, P., Levine, S., and van de Panne, M. (2018). DeepMimic: Example-guided deep reinforcement learning of physics-based character skills. *ACM Transactions on Graphics* 37, 1–14. doi:10.1145/3197517.3201311

Peng, X. B., Guo, Y., Halper, L., Levine, S., and Fidler, S. (2022). ASE: Large-Scale Reusable Adversarial Skill Embeddings for Physically Simulated Characters. *ACM Transactions on Graphics* 41, 1–17. doi:10.1145/3528223.3530110

Schulman, J., Wolski, F., Dhariwal, P., Radford, A., and Klimov, O. (2017). Proximal Policy Optimization Algorithms. *arXiv:1707.06347 [cs]*

Virtanen, P., Gommers, R., Oliphant, T. E., Haberland, M., Reddy, T., Cournapeau, D., et al. (2020). SciPy 1.0: Fundamental Algorithms for Scientific Computing in Python. *Nature Methods* 17, 261–272. doi:10.1038/s41592-019-0686-2
